# Syndecan-2 regulates PAD2 to exert antifibrotic effects on RA-ILD fibroblasts

**DOI:** 10.1038/s41598-022-06678-7

**Published:** 2022-02-18

**Authors:** Konstantin Tsoyi, Anthony J. Esposito, Bo Sun, Ryan G. Bowen, Kevin Xiong, Fernando Poli, Rafael Cardenas, Sarah G. Chu, Xiaoliang Liang, Stefan W. Ryter, Christine Beeton, Tracy J. Doyle, Matthew J. Robertson, Lindsay J. Celada, Freddy Romero, Souheil Y. El-Chemaly, Mark A. Perrella, I.-Cheng Ho, Ivan O. Rosas

**Affiliations:** 1 Section of Pulmonary, Critical Care, and Sleep Medicine, Department of Medicine, Baylor College of Medicine, 7200 Cambridge Street, Houston, TX 77030 USA; 2 Division of Pulmonary and Critical Care Medicine, Department of Medicine, Brigham and Women’s Hospital, Harvard Medical School, Boston, MA USA; 3 Division of Pulmonary and Critical Care Medicine, Department of Medicine, Northwestern University Feinberg School of Medicine, Chicago, IL USA; 4 Division of Rheumatology, Inflammation, and Immunity, Department of Medicine, Brigham and Women’s Hospital, Harvard Medical School, Boston, MA USA; 5 Division of Pulmonary and Critical Care Medicine, Department of Medicine, Weill Cornell Medicine, New York, NY USA; 6 Department of Molecular Physiology and Biophysics, Baylor College of Medicine, Houston, TX USA

**Keywords:** Respiratory tract diseases, Rheumatoid arthritis, Pathogenesis

## Abstract

Rheumatoid arthritis (RA)-associated interstitial lung disease (RA-ILD) is the most common pulmonary complication of RA, increasing morbidity and mortality. Anti-citrullinated protein antibodies have been associated with the development and progression of both RA and fibrotic lung disease; however, the role of protein citrullination in RA-ILD remains unclear. Here, we demonstrate that the expression of peptidylarginine deiminase 2 (PAD2), an enzyme that catalyzes protein citrullination, is increased in lung homogenates from subjects with RA-ILD and their lung fibroblasts. Chemical inhibition or genetic knockdown of PAD2 in RA-ILD fibroblasts attenuated their activation, marked by decreased myofibroblast differentiation, gel contraction, and extracellular matrix gene expression. Treatment of RA-ILD fibroblasts with the proteoglycan syndecan-2 (SDC2) yielded similar antifibrotic effects through regulation of PAD2 expression, phosphoinositide 3-kinase/Akt signaling, and Sp1 activation in a CD148-dependent manner. Furthermore, SDC2-transgenic mice exposed to bleomycin-induced lung injury in an inflammatory arthritis model expressed lower levels of PAD2 and were protected from the development of pulmonary fibrosis. Together, our results support a SDC2-sensitive profibrotic role for PAD2 in RA-ILD fibroblasts and identify PAD2 as a promising therapeutic target of RA-ILD.

## Introduction

Rheumatoid arthritis (RA) is a destructive, systemic, inflammatory disease that affects approximately 1% of the population in developed countries^1–3^. Interstitial lung disease (ILD), represented primarily by pulmonary fibrosis, is a common extra-articular manifestation of RA that occurs predominantly in older men with more severe inflammatory disease^4,5^. RA-ILD contributes to 13% of the excess mortality observed in the RA population, translating to a risk of death for individuals with RA-ILD that is three times higher than for RA patients without ILD^6–8^. Despite the devastating impact of RA-ILD, little is known about its pathophysiology. Understanding the molecular mechanisms that underlie the development and progression of RA-ILD may help to identify new targets for therapeutic development, an unmet need for this disease.

Citrullination may contribute to the development of RA, as anti-citrullinated protein antibodies (ACPA) can be detected in about 50–60% of patients with this disease^1,9^. In some instances, ACPA are detected before any signs of joint inflammation^10,11^, suggesting that RA autoimmunity may be initiated elsewhere^12^. Our group and others have previously shown that ACPA titers are significantly higher in patients with RA-ILD compared to RA without ILD^13–15^, proposing that aberrant citrullination may contribute to the pathogenesis of pulmonary fibrosis in RA patients. Protein citrullination is catalyzed by peptidylarginine deiminases (PADs) which convert peptidylarginine to peptidylcitrulline in the presence of calcium^16,17^. PAD2 (gene symbol: *PADI2)* is highly expressed in various cell types including immune, endothelial, and fibroblast-like cells of synovial tissue from RA patients^16,18,19^. Interestingly, PAD2 has been recently shown to be involved in the pathogenesis of idiopathic pulmonary fibrosis (IPF) by a mechanism that is independent of ACPA^20^. Although certain forms of RA-ILD mimic the phenotype of IPF^5,21,22^, the role of PAD2 in the pathogenesis of RA-ILD has yet to be determined.

PAD2 gene expression is dependent on the transcription factor Sp1^23^, which in turn is regulated by phosphoinositide 3-kinase (PI3K)/Akt signaling^24–26^. Exposure to cadmium and carbon black in cigarette smoke has been shown to induce secretion of citrullinated vimentin (cit-vimentin) in an Akt- and PAD2-dependent manner, evoking a profibrotic phenotype in fibroblasts^20^. Our laboratory has previously demonstrated that inhibition of PI3K/Akt signaling by the heparin-sulfate proteoglycan syndecan-2 (SDC2) has a potent antifibrotic effect in transforming growth factor beta-1 (TGF-β1)-activated lung fibroblasts via activation of the receptor-like protein tyrosine phosphatase eta (PTPRj/CD148)^27,28^. We thus hypothesized that PAD2 contributes to the pathogenesis of RA-ILD by inducing a profibrotic phenotype in fibroblasts that is abrogated by SDC2 via CD148-dependent inhibition of PI3K/Akt/Sp1 signaling. Here, we report that PAD2 expression is increased in the lungs from subjects with RA-ILD and mediates profibrotic processes—such as myofibroblast differentiation, gel contraction, and extracellular matrix production—in their lung fibroblasts. We demonstrate that SDC2 downregulates PAD2 expression and attenuates fibroblast activation by inhibiting the PI3K/Akt/Sp1 signaling pathway via CD148. Finally, we demonstrate that SDC2-transgenic mice express lower levels of PAD2 and are protected from lung fibrosis after bleomycin-induced lung injury in a model of inflammatory arthritis.

## Results

### PAD2 Levels are Elevated in RA-ILD Lung and Associated Fibroblasts

**Figure 1:**
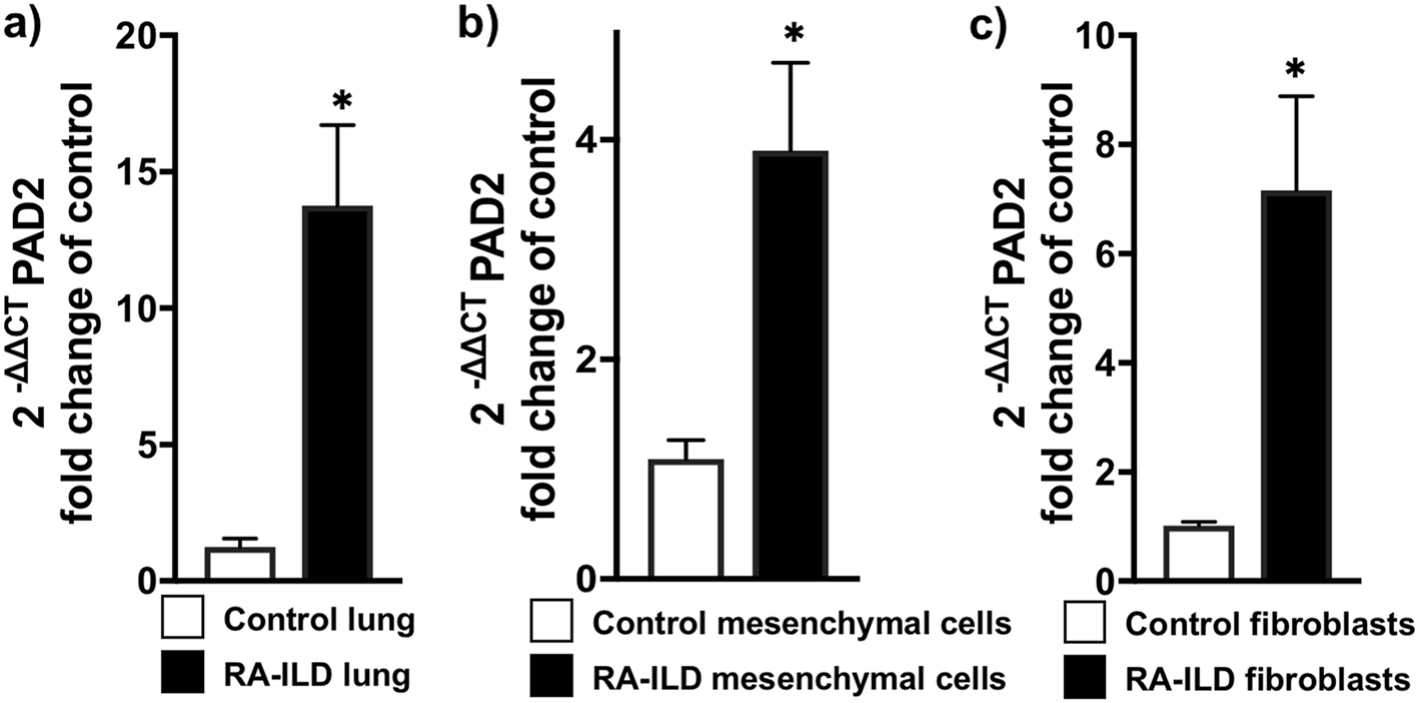
PAD2 is upregulated in RA-ILD fibroblasts. (**A**) Control (*n* = 4) and RA-ILD (*n* = 6) lungs were digested and subjected to total RNA isolation. mRNA levels of PAD2 were measured by RT-PCR. (**B**) Sorted mesenchymal (Mes) cells from control (*n* = 6) and RA-ILD (*n* = 6) lungs were subjected to total RNA isolation. mRNA levels of PAD2 were measured by RT-PCR as described in the Methods. (**C**) Primary human lung fibroblasts from control (*n* = 4) and RA-ILD (*n* = 4) lungs were isolated as described in the Methods. Data are mean ± SEM. *P* < 0.05; significant comparisons Student’s unpaired t test: *vs. control. Abbreviations: ILD = interstitial lung disease, PAD2 = peptidylarginine deminase 2, RA = rheumatoid arthritis, RT-PCR = real-time polymerase chain reaction.

PADs are known to play a critical role in the pathogenesis of RA^19,29–32^; however, knowledge about their role in pulmonary fibrosis, particularly RA-ILD, is limited^20,33^. Since fibroblasts are the effector cells of organ fibrogenesis, we assessed the genetic expression of various PADs in RA-ILD lung tissue, sorted mesenchymal cells (an enriched fibroblast population), and primary cultured fibroblasts by real-time polymerase chain reaction (RT-PCR). As shown in Fig. 1A, *PADI2* expression was significantly elevated in RA-ILD lung tissue homogenate compared to control lung. Mesenchymal cells were sorted from control and RA-ILD lung as previously described^34,35^. We found that *PADI2* expression was also elevated in sorted mesenchymal cells and cultured fibroblasts derived from RA-ILD lung (Fig. 1B,C). Of note, we observed a mild increase in *PADI4* expression in RA-ILD lung but not in isolated fibroblasts (Supplementary Fig. S1A and S1B online); however, no difference in *PADI1*, *PADI3*, and *PADI6* expression was observed between RA-ILD and control lung (Supplementary Fig. S1A online).

### PAD2 promotes a profibrotic phenotype in RA-ILD fibroblasts

**Figure 2:**
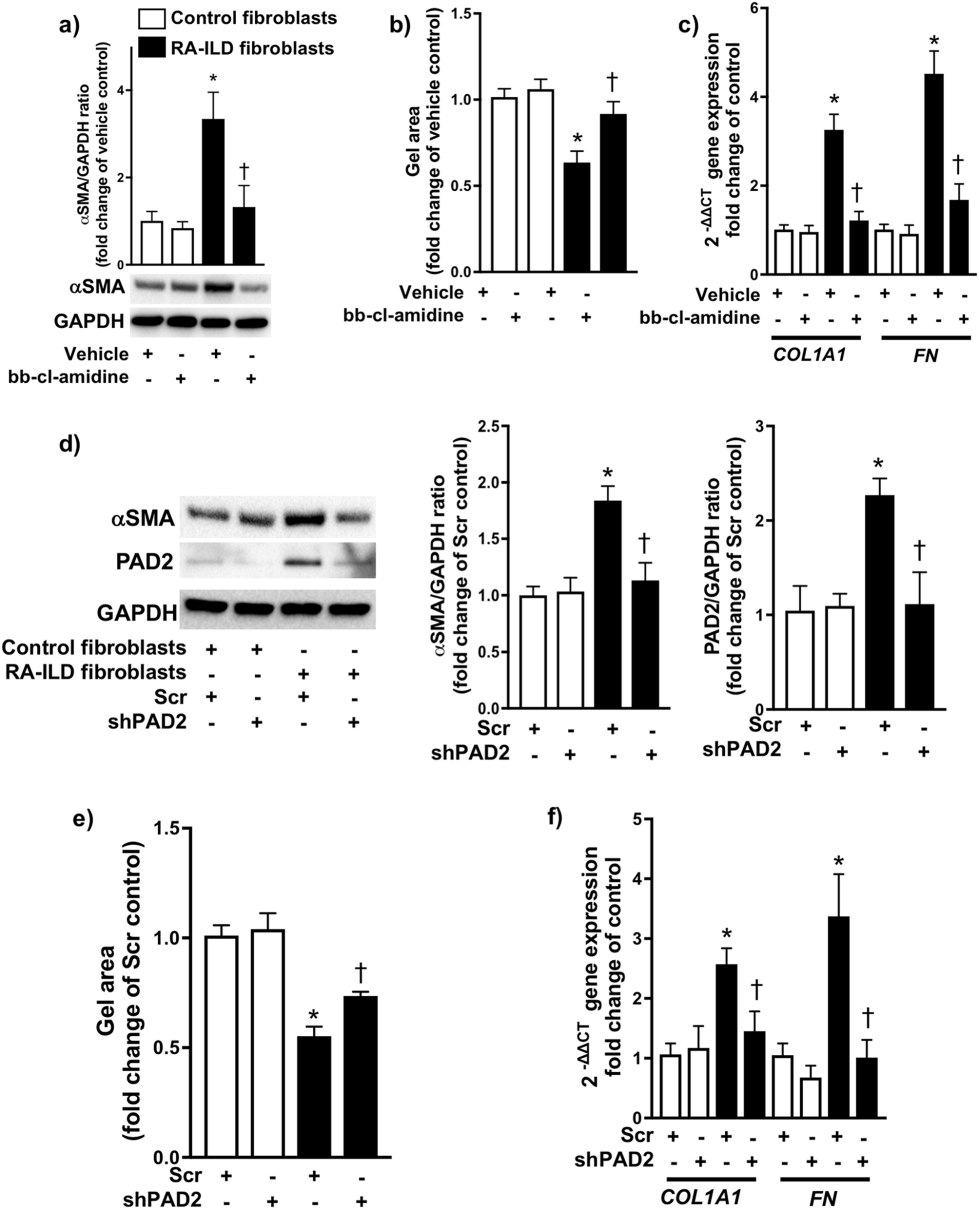
PAD2 regulates myofibroblast differentiation and extracellular matrix production. (**A**) Control (*n* = 4) and RA-ILD (*n* = 4) fibroblasts were treated with vehicle or bb-chloride-amidine (1 μM). 24 h later, cells were lysed and subjected to Western blot analysis to measure α-SMA expression. (**B**) Cells were mixed with collagen 1 solution as described in the Methods. Gel size was measured at 0 and 24 h after collagen gelation (*n* = 4 for each condition). (**C**) Cells were treated as described in (A). After treatment, cells were lysed for total RNA isolation. mRNA levels of collagen1a1 (*COL1A1*) and fibronectin (*FN*) were measured by RT-PCR. (**D-E**) Control (*n* = 4) and RA-ILD (*n* = 4) fibroblasts were transfected with scrambled (scr) or shPAD2 via lentivirus as described in the Methods. Stably transfected cells were subjected to Western blot analysis to measure (**D**) α-SMA and PAD2 expression, (**E**) cell contraction, and (**F**) RT-PCR to measure *COL1A1* and *FN*. Data are mean ± SEM. *P* < 0.05; significant comparisons by one-way ANOVA: *vs. control, ^†^*vs.* RA-ILD fibroblasts treated with vehicle or Scr. Abbreviations: α-SMA = alpha smooth muscle actin, GAPDH = glyceraldehyde 3-phosphate dehydrogenase, ILD = interstitial lung disease, PAD2 = peptidylarginine deminase 2, RA = rheumatoid arthritis, RT-PCR = real-time polymerase chain reaction, scr = scrambled short hairpin RNA, shPAD2 = PAD2 short hairpin RNA.

Next, we examined whether pharmacological inhibition and genetic knockdown of PAD2 modulated the profibrotic phenotype of RA-ILD fibroblasts. Administration of a non-selective PAD inhibitor (bb-chloride-amidine) significantly decreased the protein expression of alpha-smooth muscle actin (α-SMA) (Fig. 2A) and reduced the gel contraction area (i.e., greater gel surface area due to reduced contraction) (Fig. 2B) of RA-ILD fibroblasts compared to vehicle-treated cells, both markers of myofibroblast differentiation. Likewise, bb-chloride-amidine attenuated the gene expression of collagen 1a1 (*COL1A1*) and fibronectin (*FN*) (Fig. 2C), canonical extracellular matrix markers of fibrosis. To confirm the role of PAD2, we transfected RA-ILD fibroblasts with small hairpin RNA (shRNA) specific for PAD2. Genetic knockdown of PAD2 in RA-ILD fibroblasts resulted in decreased α-SMA protein expression (Fig. 2D), gel contractility which led to an increase in gel area (Fig. 2E), and *COL1A1* and *FN* expression (Fig. 2F) compared to scrambled shRNA (Scr)-transfected cells. We also observed that silencing of PAD2 significantly decreased cit-vimentin levels without affecting total vimentin levels (Supplementary Fig. S2 online). Taken together, these data demonstrate that inhibition of PAD2 in RA-ILD fibroblasts attenuates their profibrotic phenotype, marked by decreased myofibroblast differentiation and extracellular matrix production.

### SDC2 downregulates PAD2 expression by regulating PI3K/Akt/Sp1 signaling via CD148 in RA-ILD fibroblasts

**Figure 3:**
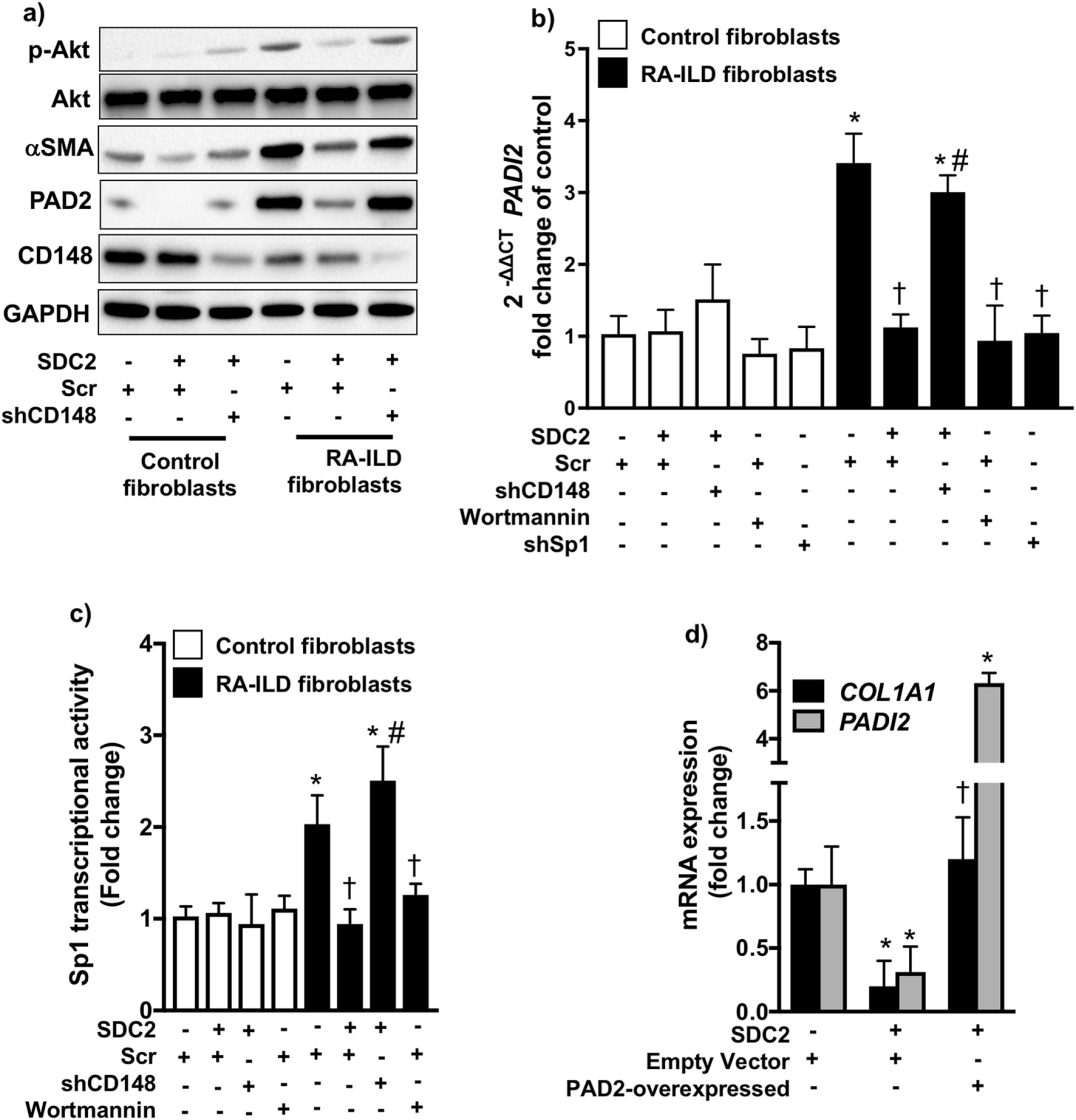
Syndecan-2 (SDC2) inhibits α-SMA and PAD2 expression by regulating CD148/PI3K/Akt signaling in RA-ILD fibroblasts. Control (*n* = 4) and RA-ILD (*n* = 4) fibroblasts were transfected with scramble (scr) or shCD148 via lentivirus as described in the Methods. Stably transfected cells were treated with syndecan-2 (10 μg/ml) for 24 h. After treatment, cells were subjected to (**A**) Western blot to measure phospho-Akt, α-SMA, and PAD2 expression, (**B**) RT-PCR to measure mRNA levels of *PADI2* (PAD2), or (**C**) a Sp1 transcriptional activity assay as described in the Methods. (**D**) RA-ILD fibroblasts were transfected via lentivirus with empty vector or pLenti-GIII-CMV-C-term-HA plasmid carrying the *PADI2* (PAD2-over). Stably transfected cells were treated with syndecan-2 (10 μM) for 24 h. After treatment, cells were subjected to RT-PCR to measure mRNA levels of *COL1A1* and *PADI2*. Data are mean ± SEM. *P* < 0.05; significant comparisons by one-way ANOVA: * *vs.* control, † *vs.* RA-ILD fibroblasts + Scr, # *vs.* RA-ILD fibroblasts + Scr + SDC2. Abbreviations: α-SMA = alpha-smooth muscle actin, ILD = interstitial lung disease, p-Akt = phosphorylated Akt, PAD2 = peptidylarginine deiminase 2, PI3K = phosphoinositide 3-kinase, RA = rheumatoid arthritis, scr = scrambled short hairpin RNA, SDC2 = syndecan-2, shCD148 = CD148 short hairpin RNA, shSp1 = Sp1 short hairpin RNA.

Sp1 is a key transcriptional activator of PAD2^23^. The transcriptional activity of Sp1 is regulated by PI3K/Akt signaling^24–26^. We have previously demonstrated that SDC2 decreases the expression of profibrotic genes in lung fibroblasts via activation of CD148, which then inhibits PI3K/Akt signaling^27,28^. Therefore, we hypothesized that SDC2 attenuates the profibrotic phenotype of RA-ILD fibroblasts by inhibiting PAD2 expression via CD148. CD148 levels tended to be lower in RA-ILD compared to control fibroblasts although the difference was not statistically significant (*p* = 0.09; Supplementary Fig. 3D online). As shown in Fig. 3A, recombinant SDC2 (10 μg/ml) significantly diminished PAD2 expression in Scr- but not shCD148-transfected cells. We also observed decreased α-SMA expression and downregulation of PI3K/Akt signaling (assessed by phosphorylated Akt levels) by SDC2 in a CD148-dependent manner (Fig. 3A and Supplementary Fig. S3 online). Overexpression of CD148 in RA-ILD fibroblasts did not significantly alter the expression of *PADI2* as measured by RT-PCR (Supplementary Fig. S4 online).

To further establish the role of PI3K/Akt/Sp1 signaling in PAD2 expression, we inhibited PI3K/Akt signaling by wortmannin (10 nM) or Sp1 expression by shRNA (Supplementary Fig. S5 online) in RA-ILD fibroblasts. As shown in Fig. 3B, both wortmannin and shSp1 significantly attenuated the expression of *PADI2*, which was similar to the effect of treatment with SDC2. We next measured the transcriptional activity of Sp1 by an enzyme-linked immunosorbent assay. We found that SDC2 inhibits Sp1 activation in a CD148-dependent manner and that wortmannin mimics the observed effect of SDC2 (Fig. 3C). Finally, to understand whether the inhibitory effect of SDC2 on PAD2 contributes to its antifibrotic effects, we overexpressed *PADI2* in RA-ILD fibroblasts in the presence of SDC2. As shown in Fig. 3D, SDC2 potently inhibited *COL1A1* expression in empty vector-transfected cells but had no effect in cells overexpressing *PADI2*. Taken together, these data suggest that SDC2 inhibits PI3K/Akt/Sp1 signaling via CD148 resulting in decreased PAD2 expression and fibroblast activation.

### Overexpression of SDC2 inhibits PAD2 and protects mice from pulmonary fibrosis in an experimental inflammatory arthritis model

Previously, we have demonstrated that the expression of SDC2 is increased in alveolar macrophages from patients with IPF^36^. We have also determined that overexpression of SDC2 in alveolar macrophages protects mice from bleomycin- and radiation-induced pulmonary fibrosis^28,36^. To study the in vivo role of SDC2 in RA-ILD, we developed a two-hit model in which we utilized the combination of collagen antibody-induced arthritis (CAIA)^37^, a previously validated mouse model of inflammatory arthritis characterized by administration of collagen type II autoantibodies and lipopolysaccharide (LPS)^38–40^, followed by lung injury with bleomycin to induce pulmonary fibrosis (Supplementary Fig. S6 online). Mice subjected to inflammatory arthritis and bleomycin-induced lung injury exhibited increased pulmonary fibrosis (Supplementary Fig. S6A online) compared to bleomycin alone or bleomycin + LPS treatment groups, suggesting that inflammatory arthritis amplifies bleomycin-induced pulmonary fibrosis. Notably, CAIA alone was insufficient to induce pulmonary fibrosis compared to saline control (Supplementary Fig. S6A online). We also observed prolonged arthritis and increased lung inflammation in mice subjected to the combination treatment compared to either condition independently (Supplementary Figs. S6B and S6C online).

**Figure 4:**
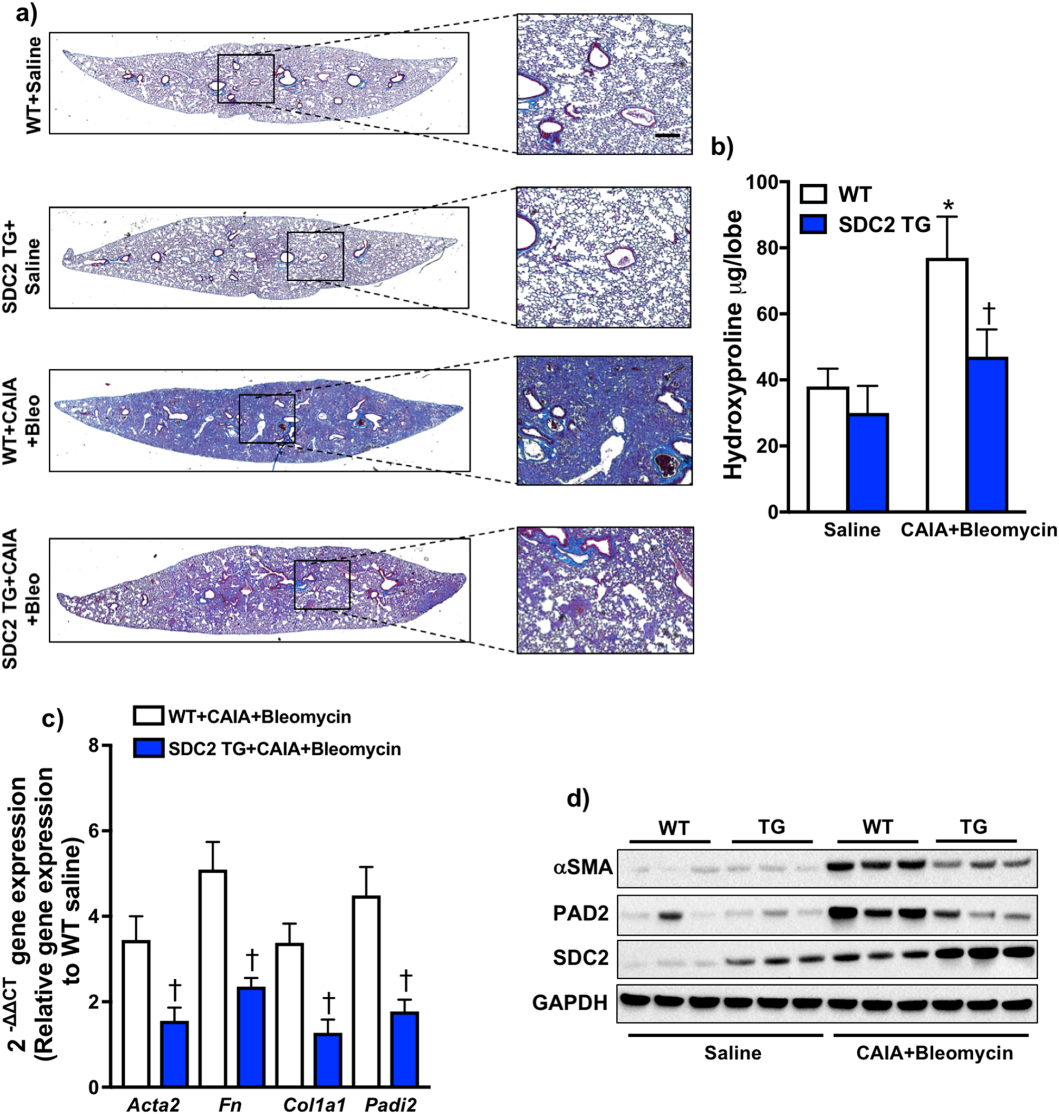
Overexpression of syndecan-2 (SDC2) attenuates fibrosis in a mouse model of pulmonary fibrosis in the setting of inflammatory arthritis. Wild type (WT) and SDC2-transgenic mice (TG) were subjected to collagen antibody-induced arthritis (CAIA) followed by bleomycin (Bleo) injury or saline as described in the Methods. (**A**) 24 days later, mouse lungs were harvested and stained with Masson’s trichrome (*n* = 3 for saline and *n* = 5 for CAIA + Bleo groups). (**B**) Hydroxyproline content was measured in the left lung of WT (*n* = 12) and SDC2 TG mice (*n* = 12) exposed to CAIA + Bleo; and WT (*n* = 5) and SDC2 TG (*n* = 5) exposed to saline. (**C**) Gene expression of *Fn* and *Col1a1*, *Acta2, Sdc2*, and *Padi2* in harvested lungs was measured using RT-PCR (*n* = 5 for each condition). (**D**) α-SMA, PAD2 and SDC2 levels were measured by Western blot. Data are mean ± SEM. *P* < 0.05; significant comparisons by one-way ANOVA: *vs. WT/saline, ^†^*vs.* WT/CAIA + Bleo alone. Abbreviations: α-SMA = alpha-smooth muscle actin, Bleo = bleomycin, CAIA = collagen antibody-induced arthritis, GAPDH = glyceraldehyde 3-phosphate dehydrogenase, PAD2 = peptidylarginine deiminase 2, SDC2 = syndecan-2, TG = transgenic, WT = wild-type.

SDC2 transgenic mice that overexpress human SDC2 in a macrophage-specific manner were subjected to CAIA followed by bleomycin-induced lung injury (CAIA + Bleo). Twenty-four days after the induction of inflammatory arthritis and twenty-one days after bleomycin injury, the lungs were harvested. Masson’s trichrome and hematoxylin and eosin staining revealed decreased collagen deposition in the lungs of SDC2 transgenic mice (Fig. 4A and Supplementary Fig. S7A online). Furthermore, the lungs of SDC2 transgenic mice had significantly less hydroxyproline levels, a constituent of collagen, compared to wild-type mice subjected to Col II ab/LPS + BLM (Fig. 4B). The expression of profibrotic genes (*Col1a1* and *Fn1*) was downregulated in the lungs of SDC2 transgenic mice compared to wildtype mice (Fig. 4C). α-SMA (*Acta2*) and PAD2 (*Padi2*) expression at the RNA (Fig. 4C) and protein level (Fig. 4D and Supplementary Fig. S7B online) were increased in the lungs of wildtype but not SDC2 transgenic mice receiving CAIA + Bleo treatment. Finally, we measured the levels of interleukin-6 (IL-6) in bronchoalveolar lavage fluid (Supplementary Fig. S8 online). Although IL-6 appeared to be lower in SDC2 transgenic mice, the difference was not significant compared to wildtype mice subjected to CAIA + Bleo. Taken together, these data demonstrate a potent antifibrotic effect of SDC2 and an associated inhibitory effect on PAD2 expression in an experimental RA-ILD mouse model.

## Discussion

Although ACPA have been associated with the development of RA-ILD^13–15^, the molecular mechanisms by which aberrant citrullination contribute to the pathogenesis of RA-ILD remain to be determined. Here, we identify a profibrotic role for the citrullinating enzyme PAD2 in fibroblasts from RA-ILD lung. Our data demonstrate that PAD2 is increased in lungs and associated fibroblasts from subjects with RA-ILD compared to control subjects and is therefore relevant to human disease. PAD2 functions as a profibrotic mediator in RA-ILD fibroblasts independent of ACPA such that its inhibition attenuates myofibroblast differentiation and extracellular matrix production. This cellular process is regulated, at least in part, by SDC2 via modulation of the PI3K/Akt/Sp1 pathway in a CD148-dependent manner. Our results further detail the importance of in vivo regulation of PAD2 by SDC2, as transgenic mice that overexpress human SDC2 displayed decreased bleomycin-induced pulmonary fibrosis and reduced PAD2 expression after bleomycin-induced lung injury in an inflammatory arthritis model. PAD2 therefore may represent a significant molecular target for the treatment of RA-ILD.

Protein citrullination has been identified as a key biochemical process in the development and progression of both RA and lung fibrosis. ACPA are positive in 50–60% of RA patients, portend a poorer prognosis, and may be detected more than ten years before joint inflammation, suggesting extra-articular generation^1,9–12^. RA-ILD is associated with the presence of ACPA^13–15^. The most common presentation of RA-ILD is of the usual interstitial pneumonia (UIP) pattern^5,21,22^, which is radiographically and histologically indistinguishable from IPF. Patients with RA-UIP tend to have different risk factors, genetic polymorphisms, and worse survival compared to RA-ILD patients with non-UIP disease that mirror those described for IPF patients^22,41,42^, suggesting mechanistic overlap between the two diseases^43,44^. Accordingly, citrullinated proteins are increased in the bronchoalveolar lavage fluid of patients with RA-ILD and IPF^44^. Furthermore, ACPA positivity has been described in a number of IPF cohorts compared to the general population and has been correlated with ectopic lymphoid aggregates in the lung^42^. Aberrant citrullination may thus represent a discrete mechanism by which fibrosis in RA-ILD and IPF develops and progresses, justifying its further investigation.

PADs, as protein citrullinating enzymes, are increasingly recognized as critical mediators of profibrotic cellular processes. Martinod et al*.* demonstrated that aged mice deficient in PAD4 were protected from the development of cardiac and pulmonary fibrosis through suppression of neutrophil extracellular trap formation^33^. Consistently, incubation of fibroblasts with PAD4-dependent neutrophil extracellular traps in vitro has been shown to induce differentiation to collagen-depositing myofibroblasts^45^. PAD2 has also been implicated in pulmonary fibrosis. For example, a recent study by Li et al*.* demonstrated that secretion of citrullinated vimentin was induced by an Akt1- and PAD2-dependent mechanism in IPF fibroblasts and that PAD2 knockout mice were protected from cadmium/carbon black (elements of cigarette smoke)-induced lung fibrosis^20^. Our data adds to this growing body of evidence. In particular, we demonstrate that PAD2 is important for the development of RA-ILD by promoting a profibrotic phenotype in RA-ILD fibroblasts, independent of ACPA, that is mitigated by treatment with SDC2. Thus, this study strongly supports that PAD2 inhibition may represent a promising strategy for the treatment of lung fibrosis.

The molecular mechanism by which PAD2 contributes to lung fibrosis remains elusive. Prior studies have suggested that PAD2 may citrullinate key signaling proteins^46–48^, which may impact their activation and/or intracellular localization. For example, PAD2 has been shown to citrullinate the gamma and p65 subunits of nuclear factor kappa-light-chain enhancer of activated B cells (NF-κB), which results in the enhancement of NF-κB activity in LPS-stimulated macrophages and neutrophils^46–48^. Alternatively, PADs can epigenetically modify profibrotic gene expression by citrullinating histones. In neutrophils, PAD4-mediated histone citrullination relaxes histone-DNA interactions, resulting in chromatin unwinding and subsequent neutrophil extracellular trap formation^49,50^. Our results and those of Li et al*.* implicate the kinase Akt in the function of PAD2 in fibroblasts from fibrotic lung diseases, leading to downstream regulation of cit-vimentin^20^. We demonstrate that inhibition of PI3K by wortmannin decreases PAD2 expression in RA-ILD fibroblasts. Furthermore, treatment of RA-ILD fibroblasts with SDC2 decreases the levels of phosphorylated Akt and PAD2 and attenuates myofibroblast differentiation and extracellular matrix gene expression. Silencing of PAD2 led to decreased expression of cit-vimentin in RA-ILD fibroblasts without affecting total levels of the vimentin protein. This latter finding is significant, as cit-vimentin has been shown to contribute to the activation of fibroblasts and development of experimental pulmonary fibrosis via a PAD2-dependent mechanism in IPF^20^. While PAD2 is likely an important downstream target of the PI3K/Akt signaling pathway, further studies are needed to establish the molecular mechanisms by which PAD2 promotes lung fibrosis.

We have previously shown that SDC2 is a potent antifibrotic proteoglycan in pulmonary fibrosis^27,28,36^. Its mechanism of action may vary depending on cell type. In alveolar epithelial cells, SDC2 has been shown to inhibit cell death induced by TGF-β1^27,28,36^. This effect was attributed to the ability of SDC2 to facilitate the internalization and subsequent degradation of TGF-β receptor I in a caveolin-1-dependent fashion. Conversely in lung fibroblasts, SDC2 does not regulate TGF-β receptor I cell surface expression but exerts its antifibrotic effect by activating CD148, leading to dephosphorylation and inactivation of PI3K/Akt signaling induced by TGF-β1^27,28^. Recently, we have extended the knowledge of the roles of SDC2 and CD148 in pulmonary fibrosis. We have demonstrated that SDC2-mediated CD148 activation inhibits experimental lung fibrosis by regulating PI3K/Akt/mammalian target of rapamycin (mTOR) signaling, thereby increasing autophagy and decreasing levels of p62 and subsequent NF-κB inactivation^27^. Here, we investigated whether SDC2-mediated inhibition of PI3K/Akt signaling affects PAD2 expression by regulating Sp1 activation. Indeed, prior reports have suggested that Akt plays an important role in Sp1 transcriptional activity in different cell types^24–26^. Sp1 has also been shown to be a critical transcriptional activator of PAD2^23^. We demonstrate that SDC2 inhibits PAD2 expression by regulating PI3K/Akt/Sp1 signaling via CD148 in RA-ILD fibroblasts.

Based on these results and our prior studies, SDC2 may represent a promising therapeutic agent for the treatment of lung fibrosis. We have previously demonstrated that SDC2 overexpression in macrophages protected mice from the development of radiation- and bleomycin-induced lung fibrosis^27,28,36^. Furthermore, we have also shown that a synthetic SDC2 peptide mimetic can activate the CD148 receptor, resulting in reduced bleomycin-induced lung fibrosis in mice and attenuated profibrotic gene expression in precision-cut lung slices derived from IPF patients^27^. This study further establishes the therapeutic role of SDC2 in a distinct experimental model. We developed a double-hit animal model of fibrotic RA-ILD by inducing pulmonary fibrosis with bleomycin in mice with active inflammatory arthritis induced by coadministration of a collagen antibody with LPS, a validated RA model known as CAIA^37^. The bleomycin model of pulmonary fibrosis is the most extensively used and reproducible model of fibrotic ILDs and was necessary as a “second hit” to the CAIA model since CAIA itself was insufficient to produce pulmonary fibrosis^51^. We found that our RA-ILD model developed prominent lung fibrosis and inflammation and displayed prolonged joint arthritis compared to the control groups (CAIA or bleomycin-induced lung injury alone). Treatment with SDC2 protected mice from the development of bleomycin-induced lung fibrosis in the inflammatory arthritis mouse model and tended to decrease bronchoalveolar fluid levels of IL-6. Although we demonstrate that the antifibrotic effects of SDC2 in vivo are associated with decreased PAD2 expression in the lung, an important consideration is that there is limited evidence that PAD2 is directly involved in the development of pulmonary fibrosis in our model. While our in vitro results strongly implicate PAD2 in the profibrotic processes of RA-ILD fibroblasts, future studies will require utilizing PAD2-selective inhibitors or PAD2-deficient mice in our CAIA + bleomycin lung injury RA-ILD model. Further preclinical investigation is needed to establish the roles of both SDC2 and PAD2 in fibrotic lung disease.

In conclusion, our findings suggest that SDC2 significantly attenuates the development of pulmonary fibrosis in an inflammatory arthritis mouse model and limits myofibroblast differentiation and extracellular matrix gene expression in RA-ILD fibroblasts. These findings extend our prior findings demonstrating that SDC2 can reduce bleomycin- and radiation-induced fibrosis and, importantly, further highlight the therapeutic potential of SDC2 in the treatment of fibrotic lung diseases^27,28^. Finally, we establish PAD2 as a profibrotic enzyme that is downregulated by SDC2 through CD148/PI3K/Akt/Sp1 signaling. These studies identify PAD2 as a promising therapeutic target in RA-ILD.

## Methods

### Human samples and ethical considerations

All human samples were obtained by Institutional Review Board-approved protocols at Brigham and Women’s Hospital (protocol number: 2011P002419) (Boston, MA) and Baylor College of Medicine (protocol number: H-46823) (Houston, TX). RA-ILD samples were obtained from explants while control lung samples without ILD were obtained from donor lungs deemed unsuitable for transplantation. All experiments were performed in accordance with relevant guidelines and regulations. Informed consent was obtained from all study participants.

### Cell culture

Fibroblasts were isolated from RA-ILD or control lung as previously described^34,35^. Briefly, lung tissue was minced and digested in collagenase buffer. Cell pellets were resuspended and cultured for 7–10 days. Cells were CD45-depleted using CD45 microbeads (Miltenyi Biotec) to remove hematopoietic cell populations. Fibroblasts were cultured in Dulbecco’s Modified Eagle Medium (Corning) containing 10% fetal bovine serum (Corning), 100 IU of penicillin and 100 μg/ml streptomycin (Corning), 292 μg/ml L-glutamine (Corning), and 100 μg/ml Primocin (InVivoGen) in humidified incubators at 37 °C and 10% CO_2_. Cells were periodically tested for Mycoplasma spp. contamination using a commercially available kit (ThermoFisher Scientific).

### RNA isolation and RT-PCR

Total RNA was isolated using the RNeasy Mini Kit (Qiagen) according to the manufacturer’s instructions. Quantitative RT-PCR with SYBR Green Master Mix (Bio-Rad Laboratories, Hercules, CA, USA) was performed using the StepOnePlus RT-PCR System (Applied Biosystems). The relative quantity of target mRNA was calculated using the delta-delta Ct method, or 2^–ΔΔCT^, and normalized using glyceraldehyde 3-phosphate dehydrogenase as an endogenous control (Sequence Detection System software, version 1.7; Applied Biosystems)^52^. Primer sequences are displayed in Supplementary Table S1 online.

### Mouse model of pulmonary fibrosis on an inflammatory arthritis background

C57BL/6 wildtype mice were purchased from Charles River Laboratories (Shrewsbury, MA). All animal experiments were approved by the Baylor College of Medicine Institutional Animal Care and Use Committee and were performed in accordance with relevant guidelines and regulations. Methods are described in accordance with ARRIVE guidelines. SDC2 transgenic mice were generated using a transgene containing the *Hsdc2* coding sequence under the control of the scavenger receptor A enhancer/promoter as described previously^28^. CAIA was induced in 8–10 week-old mice by intraperitoneal delivery of collagen type II autoantibodies (Arthritomab, BD BioSciences) at 4 mg/mouse, followed by LPS (50 μg/mouse) stimulation three days later^37^. Pulmonary fibrosis was induced by intratracheal injection of a single dose of 0.75 mg/kg body weight of bleomycin (Cayman Chemical, Ann Arbor, MI) three days after collagen type II autoantibody administration. Control mice received an equal volume of sterile saline.

### Hydroxyproline assay

To quantify collagen deposition, the left lung from each mouse was hydrolyzed in 6 N HCl for 24 h at 110 °C, and hydroxyproline levels were quantified as previously described^28,36^. Each sample was assayed in triplicate. Data are reported as micrograms of hydroxyproline per left lung.

### Histology

Lung sections were fixed by inflation with buffered 10% formalin solution and embedded in paraffin. Thin (4 μm) sections were deparaffinized and rehydrated. Sections were then stained with hematoxylin–eosin and Masson's trichrome to evaluate histopathologic changes and collagen deposition, respectively^28^.

### Lentiviral transfection

PLKO.1 plasmids carrying either the human PTPRj/CD148 shRNA target sequence.

ACGAGTCGTCATCTAACTATA (consortium number TRCN0000320555), shSp1 target sequence GCTGGTGGTGATGGAATACAT (consortium number TRCN0000020448), shPAD2 target sequence ACACCGTGATATTCCGGATTG (consortium number TRCN0000422076), or a Scr sequence were purchased from Sigma-Aldrich (St Louis, MO). Lentiviral particles were generated by use of a commercially available packaging mix (Cat. #: SHP001; Sigma-Aldrich, St Louis, MO) in human embryonic kidney 293 T cells according to the manufacturer's instructions. Cells were infected with the lentiviral particles and stably selected by use of puromycin (10 μg/mL). To overexpress human PAD2, pLenti-GIII-CMV-C-term-HA plasmid carrying the PAD2 open reading frame (ABMgood, Richmond, BC, Canada) were stably transfected by lentiviral particles generated from a commercially available packaging kit (Cat. #: LV098; ABMgood, Richmond, BC, Canada) in human embryonic kidney 293 T cells according to the manufacturer's instructions.

### Sp1 transcriptional assay

Sp1 transcriptional activity was measured using a commercially available kit from RayBiotech (Peachtree Corners, GA) according to the manufacturer’s instructions.

### Western blot

Polyacrylamide gel electrophoresis and immunoblotting were performed according to standard methods as previously described^53^. Quantification of immunoreactive protein bands was performed with the computer software ImageJ (Image Processing and Analysis in Java Edition: 1.29, URL: http://rsb.info.nih.gov/ij/ NIH, Bethesda, Maryland) and is expressed as a ratio of band intensity with respect to the loading control. Original uncropped and unadjusted Western blots are displayed in the Supplementary Information online. Antibodies to α-SMA and GAPDH were obtained from Abcam (Cambridge, MA); SDC2 from Santa Cruz Biotechnology (Santa Cruz, CA); PAD2, phospho-PI3K (p-p85), and phospho-Akt from Cell Signaling Technology (Berkeley, CA); cit-vimentin and vimentin from Cayman Chemicals (Ann Arbor, MI); and CD148 and Sp1 from R&D systems (Minneapolis, MN).

### Cell contraction assay

Control and RA-ILD fibroblast pellets were mixed with eight volumes of rat tail type I collagen suspension, one volume of 10 × concentrated PBS, and one volume of reconstitution buffer (2% sodium bicarbonate and 4.77% HEPES dissolved in 0.05 N NaOH) at a concentration of 2 × 10^6^ cells/ml. Cell-populated collagen solution was immediately poured into a 24-well-plate (0.5 ml/well) and incubated at 37 °C for one hour to permit complete gelation. After gelation, gels were gently released from the plate with a spatula and overlaid with culture media. Gels were measured with a ruler at 0 and 24 h.

### Statistics

Data are expressed as mean ± standard error the mean. For comparisons between two groups, we used Student’s unpaired t test. One-way analysis of variance followed by Newman-Keuls or Tukey’s post-test analysis was used for analysis of more than two groups. The numbers of samples per group (n), or the numbers of experiments, are specified in the figure legends. Statistical significance was defined as p < 0.05.

## Supplementary Information

Supplementary Information.

## Data Availability

The data generated and/or analyzed during the current study are available from the corresponding author on reasonable request.

## References

[1] Aletaha D (2010). 2010 Rheumatoid arthritis classification criteria: An American College of Rheumatology/European League Against Rheumatism collaborative initiative. Arthritis Rheum..

[2] Gabriel SE, Crowson CS, O'Fallon WM (1999). The epidemiology of rheumatoid arthritis in Rochester, Minnesota, 1955–1985. Arthritis Rheum..

[3] Alamanos Y, Voulgari PV, Drosos AA (2006). Incidence and prevalence of rheumatoid arthritis, based on the 1987 American College of Rheumatology criteria: A systematic review. Semin. Arthritis. Rheum..

[4] Kelly CA (2014). Rheumatoid arthritis-related interstitial lung disease: Associations, prognostic factors and physiological and radiological characteristics–a large multicentre UK study. Rheumatology (Oxford).

[5] Esposito AJ, Chu SG, Madan R, Doyle TJ, Dellaripa PF (2019). Thoracic manifestations of rheumatoid arthritis. Clin. Chest Med..

[6] Minaur NJ, Jacoby RK, Cosh JA, Taylor G, Rasker JJ (2004). Outcome after 40 years with rheumatoid arthritis: A prospective study of function, disease activity, and mortality. J. Rheumatol. Suppl..

[7] Turesson C, O'Fallon WM, Crowson CS, Gabriel SE, Matteson EL (2003). Extra-articular disease manifestations in rheumatoid arthritis: Incidence trends and risk factors over 46 years. Ann. Rheum. Dis..

[8] Bongartz T (2010). Incidence and mortality of interstitial lung disease in rheumatoid arthritis: A population-based study. Arthritis Rheum..

[9] Nishimura K (2007). Meta-analysis: diagnostic accuracy of anti-cyclic citrullinated peptide antibody and rheumatoid factor for rheumatoid arthritis. Ann. Intern. Med..

[10] Nielen MM (2004). Specific autoantibodies precede the symptoms of rheumatoid arthritis: A study of serial measurements in blood donors. Arthritis Rheum..

[11] Rantapaa-Dahlqvist S (2003). Antibodies against cyclic citrullinated peptide and IgA rheumatoid factor predict the development of rheumatoid arthritis. Arthritis Rheum..

[12] Catrina AI, Svensson CI, Malmstrom V, Schett G, Klareskog L (2017). Mechanisms leading from systemic autoimmunity to joint-specific disease in rheumatoid arthritis. Nat. Rev. Rheumatol..

[13] Doyle TJ (2015). Detection of rheumatoid arthritis-interstitial lung disease is enhanced by serum biomarkers. Am. J. Respir. Crit. Care Med..

[14] Cavagna L (2013). The multifaceted aspects of interstitial lung disease in rheumatoid arthritis. Biomed. Res. Int..

[15] Yang JA (2019). Clinical characteristics associated with occurrence and poor prognosis of interstitial lung disease in rheumatoid arthritis. Kor. J. Intern. Med..

[16] Vossenaar ER, Zendman AJ, van Venrooij WJ, Pruijn GJ (2003). PAD, a growing family of citrullinating enzymes: Genes, features and involvement in disease. BioEssays.

[17] Suzuki A, Yamada R, Yamamoto K (2007). Citrullination by peptidylarginine deiminase in rheumatoid arthritis. Ann. N. Y. Acad. Sci..

[18] Takahara H (1989). Peptidylarginine deiminase of the mouse: Distribution, properties, and immunocytochemical localization. J. Biol. Chem..

[19] Foulquier C (2007). Peptidyl arginine deiminase type 2 (PAD-2) and PAD-4 but not PAD-1, PAD-3, and PAD-6 are expressed in rheumatoid arthritis synovium in close association with tissue inflammation. Arthritis Rheum..

[20] Li FJ (2021). Citrullinated vimentin mediates development and progression of lung fibrosis. Sci. Transl. Med..

[21] Lee HK (2005). Histopathologic pattern and clinical features of rheumatoid arthritis-associated interstitial lung disease. Chest.

[22] Kim EJ (2010). Usual interstitial pneumonia in rheumatoid arthritis-associated interstitial lung disease. Eur. Respir. J..

[23] Dong S (2005). Regulation of the expression of peptidylarginine deiminase type II gene (PADI2) in human keratinocytes involves Sp1 and Sp3 transcription factors. J. Invest. Dermatol..

[24] Liu Y, Liao R, Qiang Z, Zhang C (2017). Pro-inflammatory cytokine-driven PI3K/Akt/Sp1 signalling and H2S production facilitates the pathogenesis of severe acute pancreatitis. Biosci. Rep..

[25] Pore N (2004). Sp1 is involved in Akt-mediated induction of VEGF expression through an HIF-1-independent mechanism. Mol. Biol. Cell..

[26] Vizcaino C, Mansilla S, Portugal J (2015). Sp1 transcription factor: A long-standing target in cancer chemotherapy. Pharmacol. Ther..

[27] Tsoyi K (2021). CD148 deficiency in fibroblasts promotes the development of pulmonary fibrosis. Am. J. Respir. Crit. Care Med..

[28] Tsoyi K (2018). Syndecan-2 attenuates radiation-induced pulmonary fibrosis and inhibits fibroblast activation by regulating PI3K/Akt/ROCK pathway via CD148. Am J Respir Cell Mol Biol..

[29] Darrah E, Andrade F (2018). Rheumatoid arthritis and citrullination. Curr. Opin. Rheumatol..

[30] Koushik S (2017). PAD4: pathophysiology, current therapeutics and future perspective in rheumatoid arthritis. Expert. Opin. Ther. Targets..

[31] Kawalkowska J (2016). Abrogation of collagen-induced arthritis by a peptidyl arginine deiminase inhibitor is associated with modulation of T cell-mediated immune responses. Sci. Rep..

[32] Willis VC (2017). Protein arginine deiminase 4 inhibition is sufficient for the amelioration of collagen-induced arthritis. Clin. Exp. Immunol..

[33] Martinod K (2017). Peptidylarginine deiminase 4 promotes age-related organ fibrosis. J. Exp. Med..

[34] Chu SG (2020). Biobanking and cryopreservation of human lung explants for omic analysis. Eur. Respir. J..

[35] Tsoyi K (2016). Carbon monoxide improves efficacy of mesenchymal stromal cells during sepsis by production of specialized proresolving lipid mediators. Crit. Care Med..

[36] Shi Y (2013). Syndecan-2 exerts antifibrotic effects by promoting caveolin-1-mediated transforming growth factor-beta receptor I internalization and inhibiting transforming growth factor-beta1 signaling. Am. J. Respir. Crit. Care Med..

[37] Khachigian LM (2006). Collagen antibody-induced arthritis. Nat. Protoc..

[38] Kagari T, Tanaka D, Doi H, Shimozato T (2003). Essential role of Fc gamma receptors in anti-type II collagen antibody-induced arthritis. J. Immunol..

[39] Hamamura K (2015). Salubrinal acts as a Dusp2 inhibitor and suppresses inflammation in anti-collagen antibody-induced arthritis. Cell Signal..

[40] Leavenworth JW, Tang X, Kim HJ, Wang X, Cantor H (2013). Amelioration of arthritis through mobilization of peptide-specific CD8+ regulatory T cells. J. Clin. Invest..

[41] Juge PA (2018). MUC5B Promoter Variant and Rheumatoid Arthritis with Interstitial Lung Disease. N. Engl. J. Med..

[42] Solomon JJ (2020). IgA antibodies directed against citrullinated protein antigens are elevated in patients with idiopathic pulmonary fibrosis. Chest.

[43] Paulin F, Doyle TJ, Fletcher EA, Ascherman DP, Rosas IO (2015). Rheumatoid arthritis-associated interstitial lung disease and idiopathic pulmonary fibrosis: shared mechanistic and phenotypic traits suggest overlapping disease mechanisms. Rev. Invest. Clin..

[44] Samara KD (2017). Upregulation of citrullination pathway: From autoimmune to idiopathic lung fibrosis. Respir. Res..

[45] Chrysanthopoulou A (2014). Neutrophil extracellular traps promote differentiation and function of fibroblasts. J. Pathol..

[46] Lee, H. J. *et al.* Peptidylarginine deiminase 2 suppresses inhibitory {kappa}B kinase activity in lipopolysaccharide-stimulated RAW 264.7 macrophages. *J. Biol. Chem*. **285**, 39655–39662 (2010). 10.1074/jbc.M110.170290 PMC3000946 20937835

[47] Stadler SC (2013). Dysregulation of PAD4-mediated citrullination of nuclear GSK3beta activates TGF-beta signaling and induces epithelial-to-mesenchymal transition in breast cancer cells. Proc. Natl. Acad. Sci. USA..

[48] Sun, B. *et al.* Citrullination of NF-kappaB p65 promotes its nuclear localization and TLR-induced expression of IL-1beta and TNFalpha. *Sci. Immunol*. **2** (2017). 10.1126/sciimmunol.aal3062 PMC5718838 28783661

[49] Fan L (2017). PADI4 epigenetically suppresses p21 transcription and inhibits cell apoptosis in fibroblast-like synoviocytes from rheumatoid arthritis patients. Int. J. Biol. Sci..

[50] Lewis HD (2015). Inhibition of PAD4 activity is sufficient to disrupt mouse and human NET formation. Nat. Chem. Biol..

[51] Liu, T., De Los Santos, F. G. & Phan, S. H. The Bleomycin Model of Pulmonary Fibrosis. *Methods Mol. Biol*. **1627**, 27–42 (2017). 10.1007/978-1-4939-7113-8_2 28836192

[52] Schmittgen TD, Livak KJ (2008). Analyzing real-time PCR data by the comparative C(T) method. Nat Protoc..

[53] Tsoyi K (2015). Elk-3 is a KLF4-regulated gene that modulates the phagocytosis of bacteria by macrophages. J Leukoc Biol..

