# Supplementary material for: Syndecan-2 regulates PAD2 to exert antifibrotic effects on RA-ILD fibroblasts
Source: Sci Rep. 2022 Feb 18;12:2847. doi: 10.1038/s41598-022-06678-7 (PMC8857282; doi:10.1038/s41598-022-06678-7)
Supplement: Supplementary file 1 — Supplementary Information. [file 41598_2022_6678_MOESM1_ESM.pdf]

SUPPLEMENTARY INFORMATION

Syndecan-2 regulates PAD2 to exert antifibrotic effects on RA-ILD fibroblasts

Konstantin Tsoyi[1]*, Anthony J. Esposito[2,3], Bo Sun[4], Ryan G. Bowen[1], Kevin Xiong[2], Fernando Poli[1], Rafael Cardenas[1], Sarah G. Chu[2], Xiaoliang Liang[1], Stefan W. Ryter[5], Christine Beeton[6], Tracy J. Doyle[2], Matthew J. Robertson[1], Lindsay J. Celada[1], Freddy Romero[1], Souheil Y. El-Chemaly[2], Mark A. Perrella[2], I-Cheng Ho[4], and Ivan O. Rosas[1]

[1]Section of Pulmonary, Critical Care, and Sleep Medicine, Department of Medicine, Baylor College of Medicine, Houston, TX, USA

[2]Division of Pulmonary and Critical Care Medicine, Department of Medicine, Brigham and Women's Hospital, Harvard Medical School, Boston, MA, USA

[3]Division of Pulmonary and Critical Care Medicine, Department of Medicine, Northwestern University Feinberg School of Medicine, Chicago, IL, USA

[4]Division of Rheumatology, Inflammation, and Immunity, Department of Medicine, Brigham and Women's Hospital, Harvard Medical School, Boston, MA, USA

[5]Division of Pulmonary and Critical Care Medicine, Department of Medicine, Weill Cornell Medicine, New York, NY, USA

[6]Department of Molecular Physiology and Biophysics, Baylor College of Medicine, Houston, TX, USA

*Corresponding Author: Konstantin Tsoyi, Ph.D., Section of Pulmonary, Critical Care, and Sleep Medicine, Department of Medicine, Baylor College of Medicine, 7200 Cambridge Street, Houston, TX 77030, Email: Konstantin.Tsoyi@bcm.edu

SUPPLEMENTARY METHODS

Arthritis Score

C57BL/6 mice were subjected to collagen antibody-induced arthritis and bleomycin-induced lung fibrosis as described in the Methods. Arthritis severity was blindly scored three times a week as previously described and scaled from 0 (no redness and swelling) to 3 (severe swelling encompassing the ankle and foot or deformity).[1,2] Each limb was graded, allowing a maximum score of 12 per mouse.

Assessment of Cytokines in Bronchoalveolar Lavage (BAL) Fluid

Isolated mouse lungs were lavaged with saline via endotracheal tube (1ml per lung). BAL fluid samples were centrifuged, and interleukin-1beta (IL-1β) and IL-6 levels were measured in 50μl aliquots of the supernatant by enzyme-linked immunosorbent assay in accordance with the manufacturer's instructions (R&D systems, catalogue numbers: MLB00C, M6000B).

CD148 overexpression in lung fibroblasts

Isolated lung fibroblasts from control and RA-ILD subjects were transfected with pLenti-GIII-CD148-HA (catalogue number: LV278210) and empty vector (pLenti-GIII-HA) plasmids (ABMgood, Vancouver, BC) via lentivirus as described previously.[3] Prior to isolation of RNA, stably transfected cells were selected by use of puromycin (10 μg/ml) for 7 days.

SUPPLEMENTARY FIGURES

a)

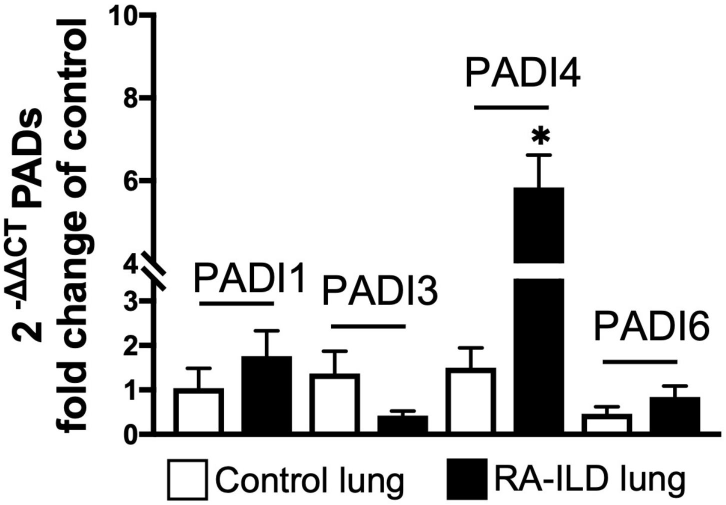

b)

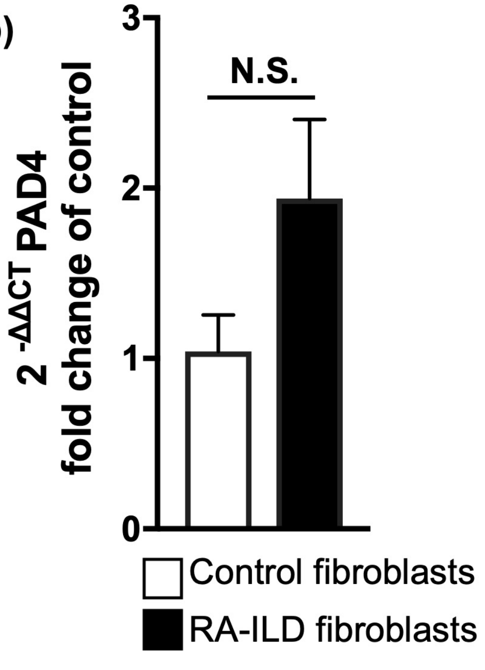

Supplementary Figure S1. The expression of PADs in RA-ILD lungs. (**A**) Control (*n*=4) and RA-ILD (*n*=6) lungs were digested and subjected to total RNA isolation. mRNA levels of *PADI1*, *PADI3*, *PADI4*, and *PADI6* were measured by RT-PCR. (**B**) Control and RA-ILD lung fibroblasts were digested and subjected to total RNA isolation. mRNA levels of *PADI4* were measured by RT-PCR. Data are mean ± SEM. $P<0.05$; significant comparisons by Student's unpaired t test. *vs. control. N.S. = not significant.

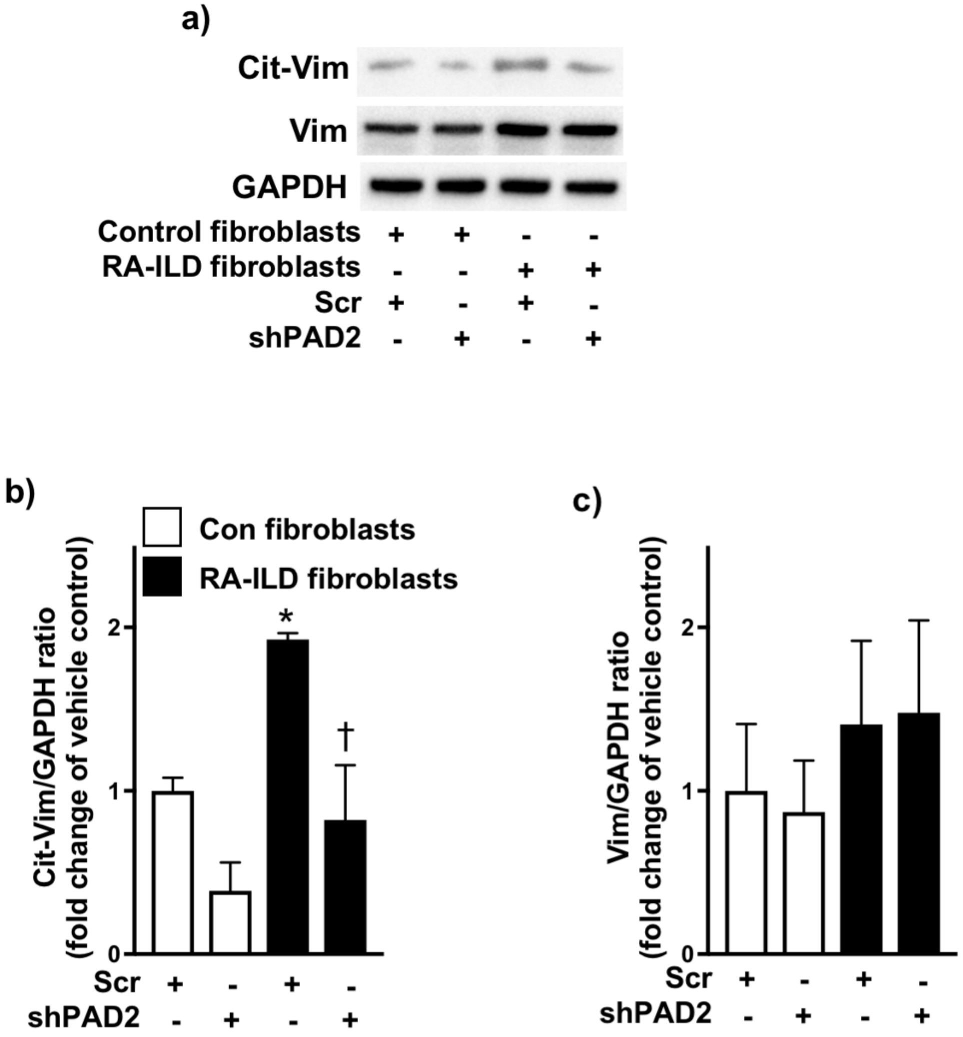

Supplementary Figure S2. PAD2 regulates citrullinated-vimentin in RA-ILD fibroblasts. Cells were transfected with scramble (Scr) or shPAD2 (n=3 per group) as described in the Methods. Cell lysates were analyzed by Western blotting to detect citrullinated-vimentin (cit-vim), vimentin, and GAPDH. Representative blots (**A**). Protein Densitometry (**B**, **C**). Data are mean ± SEM. P<0.05; significant comparisons by one-way ANOVA: **vs.* Control fibroblasts+Scr, †*vs.* RA-ILD fibroblasts+Scr.

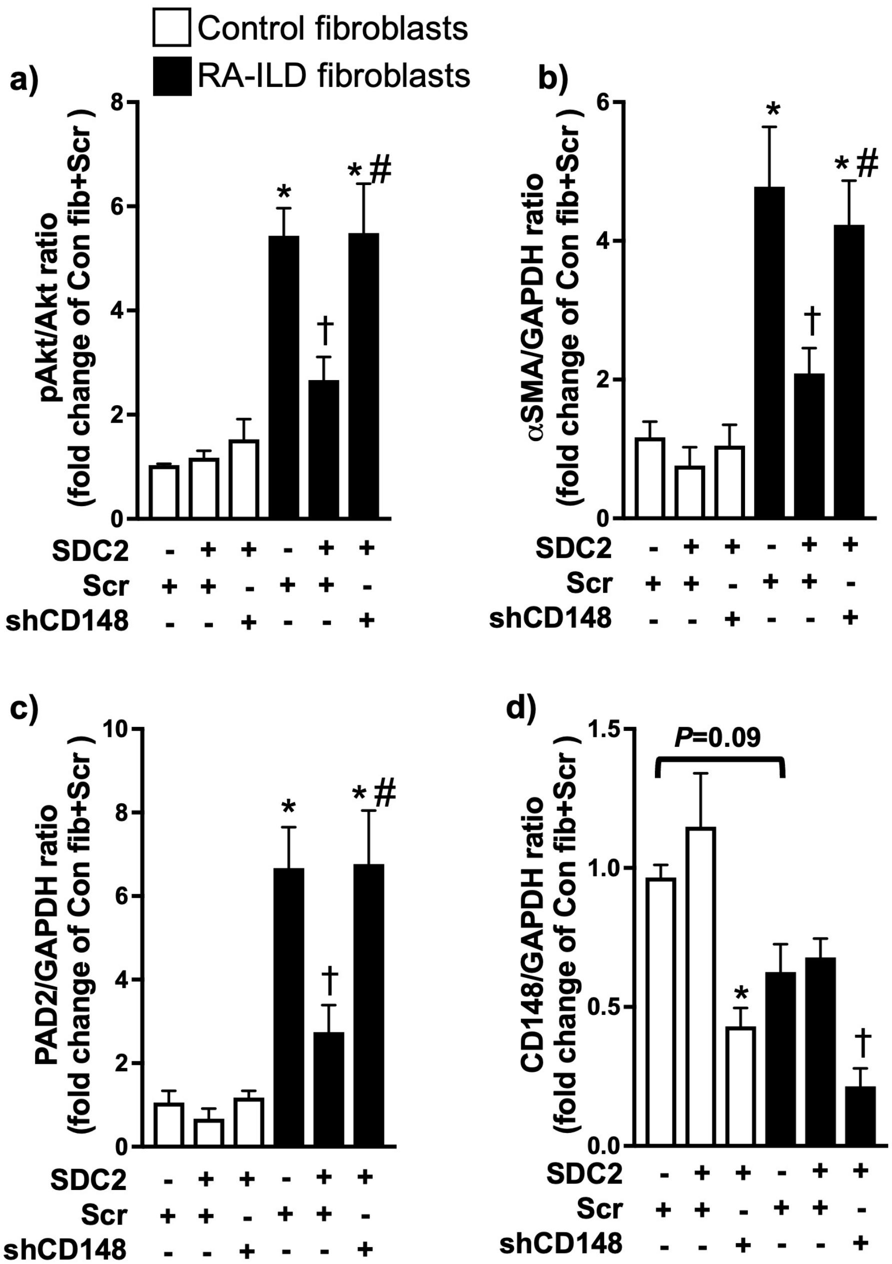

Supplementary Figure S3. Protein Densitometry. Quantification of band intensity of target proteins phospho-Akt (**A**), α-SMA (**B**), PAD2 (**C**), and CD148 (**D**) (corresponding to Western blots in Fig. 3A). Band intensities were quantified using ImageJ software and were depicted as the fold change ratio of band intensity to GAPDH. Data are mean ± SEM. $P<0.05$; significant comparisons by one-way ANOVA: **vs.* Control fibroblasts+Scr, †*vs.* RA-ILD fibroblasts+Scr, #*vs.* RA-ILD fibroblasts+Scr+SDC2.

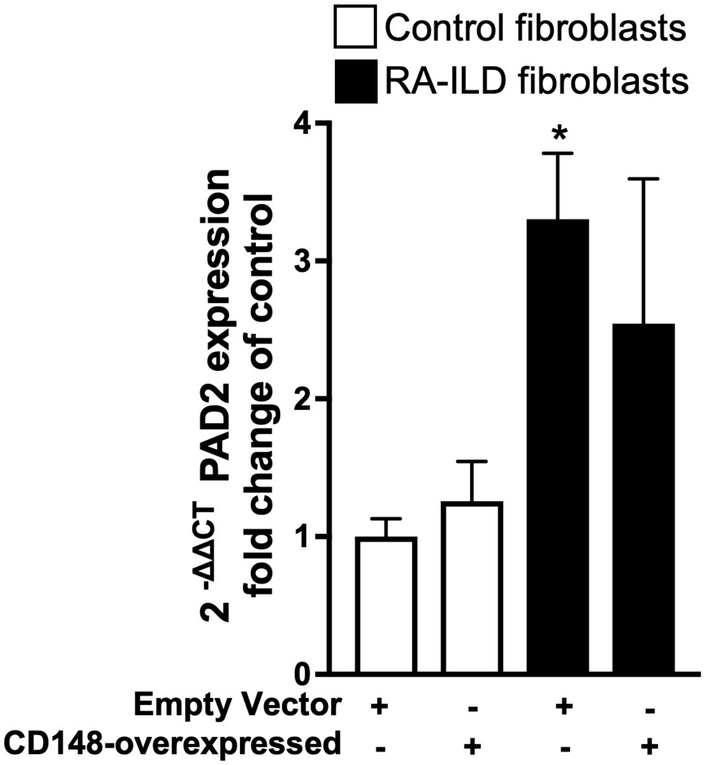

Supplementary Figure S4. CD148 overexpression does not regulate *PADI2* mRNA levels in RA-ILD fibroblasts. Cells were transfected via lentivirus with empty vector or a CD148 overexpressing vector (pLenti-GIII-CD148-HA) as described in the Methods. Cells were then lysed with Trizol to isolate total RNA. The mRNA levels of *PADI2* were measured by RT-qPCR. $P<0.05$; significant comparisons by one-way ANOVA: **vs.* control fibroblasts+Empty Vector.

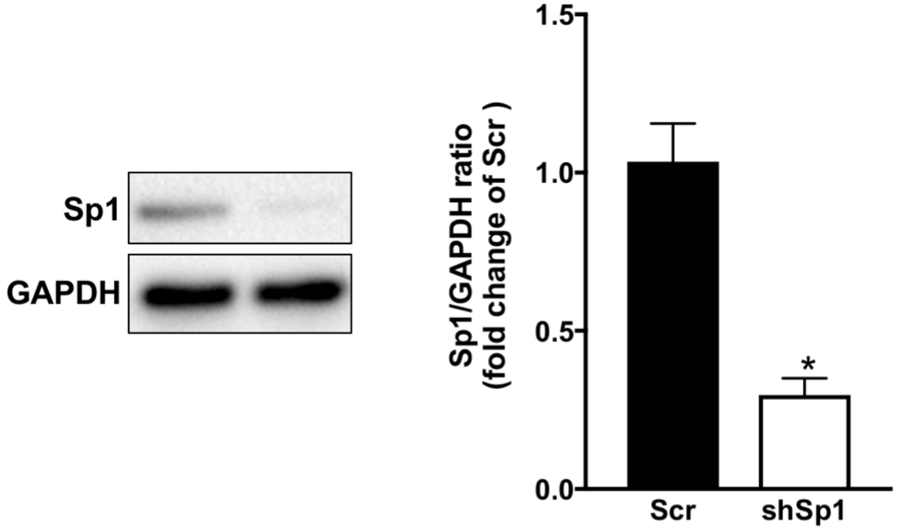

Supplementary Figure S5. shSp1 downregulates Sp1 expression in RA-ILD fibroblasts. Control (*n*=4) and RA-ILD (*n*=4) fibroblasts were transfected with scramble (Scr) or shSp1 using lentivirus as described in the Methods. Protein levels of Sp1 expression were measured by densitometry from Western blotting. Data are mean ± SEM. $P<0.05$; significant comparisons using Student's unpaired t test. **vs.* Scr.

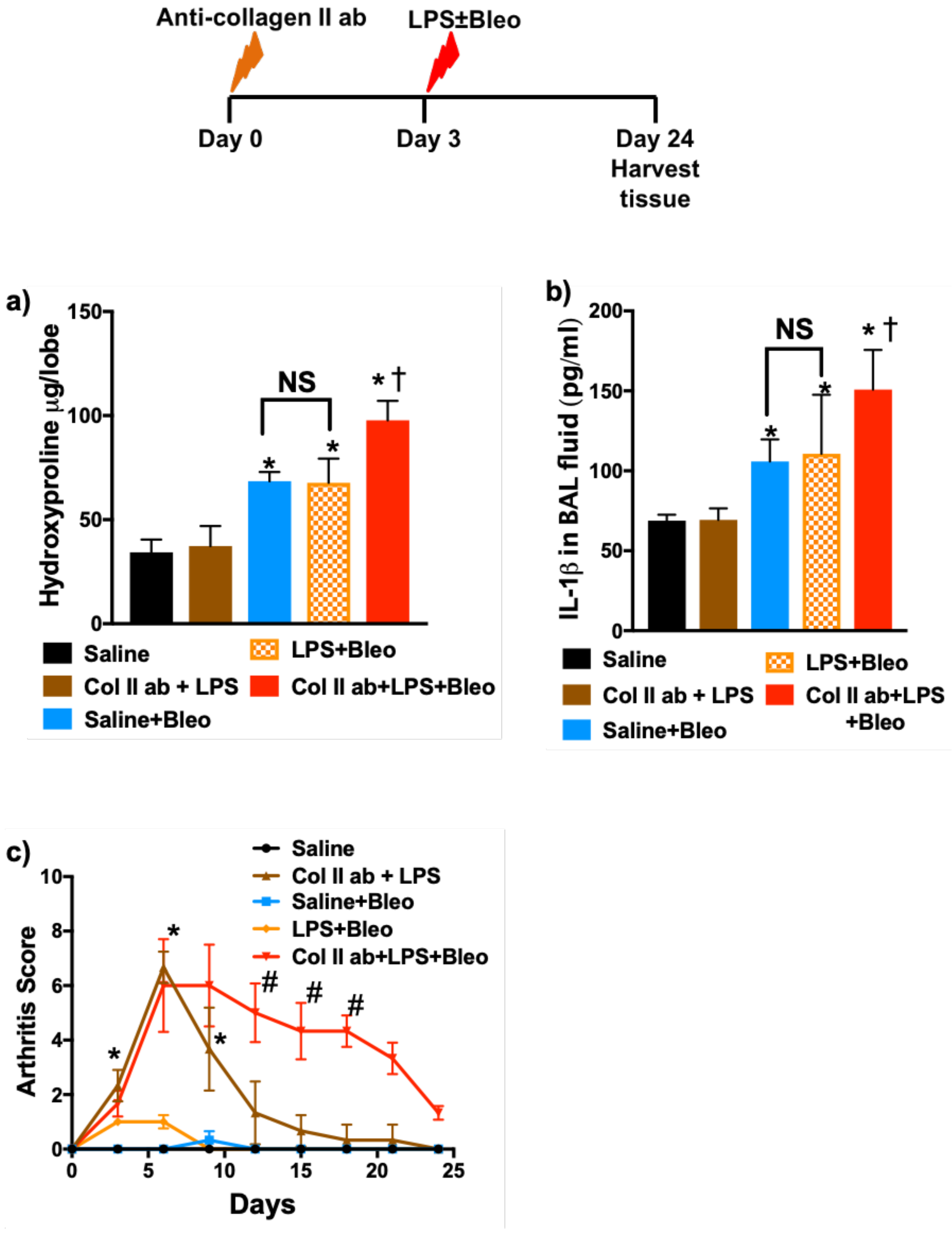

Supplementary Figure S6. Mouse model of pulmonary fibrosis in the setting of inflammatory arthritis. (**A-C**) Mice (*n*=7 per Saline group, *n*=10 per CAIA+Bleo group) were injected with mouse anti-collagen II antibodies (4 mg/mouse, *i.p.*) followed by lipopolysaccharide (50 µg/mouse, *i.p.*) induce inflammatory arthritis. Bleomycin (1 mg/kg) was intratracheally delivered to induce lung fibrosis. Hydroxyproline levels were measured at day 24 after arthritis initiation (**A**). IL-1β protein level in bronchoalveolar fluid (**B**). Arthritis score (**C**). $P<0.05$; significant comparisons: **vs.* saline, †*vs.* Bleo alone, #*vs.* CAIA. Bleo = bleomycin. CAIA =

collagen antibody-induced arthrtitis (anti-collagen II antibodies + LPS). LPS = lipopolysaccharide. N.S. = not significant.

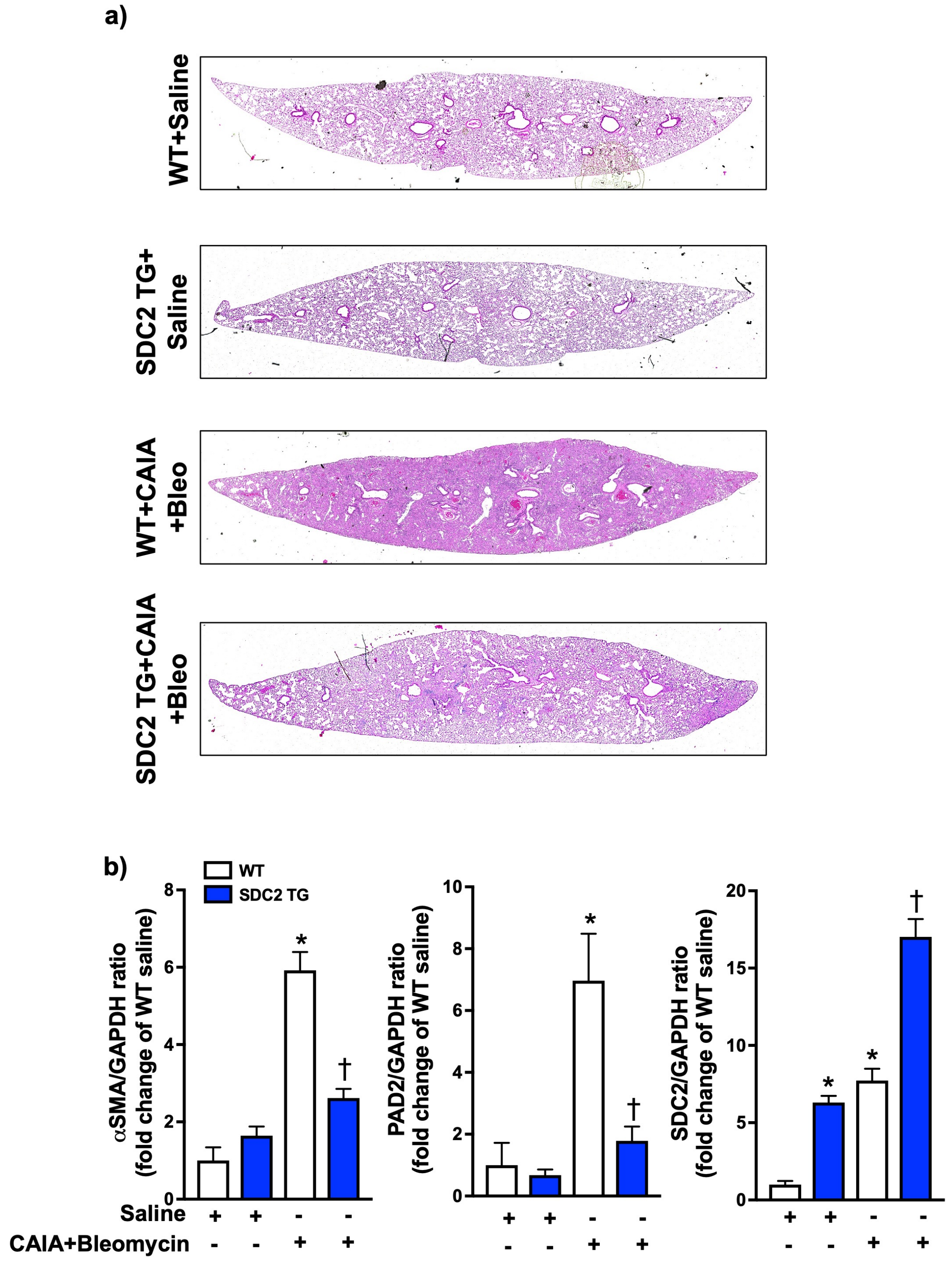

Supplementary Figure S7. H&E staining and protein densitometry of lung tissue from wild-type (WT) and syndecan-2 transgenic (SDC2 TG) mice subjected to collagen antibody-induced arthritis (CAIA) followed by bleomycin (Bleo) lung injury. (**A**) Mouse lungs were harvested and stained with hematoxylin and eosin on the same area shown for Trichrome staining in Fig. 4A. (**B**) Quantification of band intensity of target proteins α-SMA, PAD2, and SDC2 (corresponding to Western blots in Fig. 4D). Band intensities were

quantified using ImageJ software and were depicted as the fold change in the ratio of band intensity to GAPDH. $P<0.05$; significant comparisons by one-way ANOVA: *vs. WT/saline, †vs. WT/CAIA+Bleomycin.

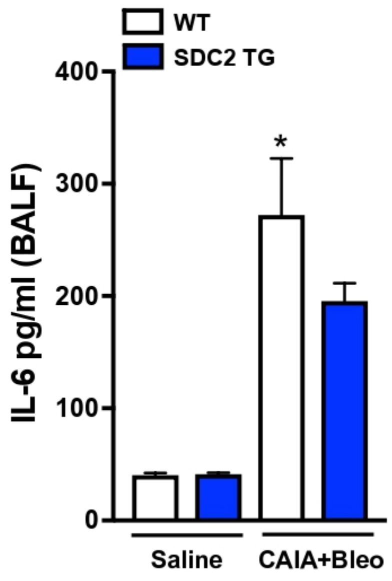

Supplementary Figure S8. Interleukin-6 expression in bronchoalveolar lavage fluid (BALF). The protein levels of IL-6 in BALF (n=5 per group) of wildtype (WT) and SDC2-transgenic (SDC2 TG) mice were measured by ELISA. *P*<0.05; significant comparisons by one-way ANOVA: **vs.* WT/saline.

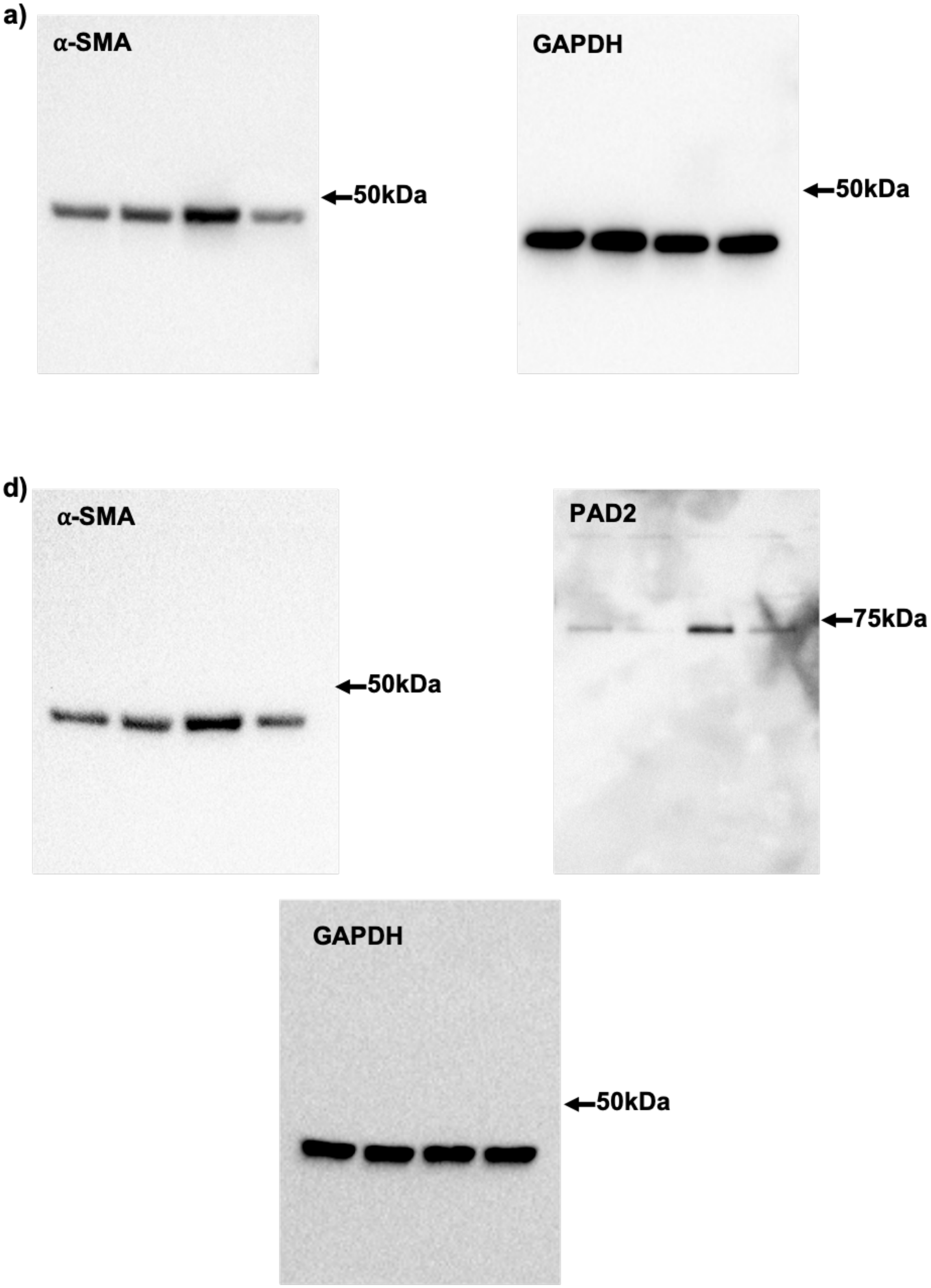

Supplementary Figure S9. Original Western blot images from Figure 2.

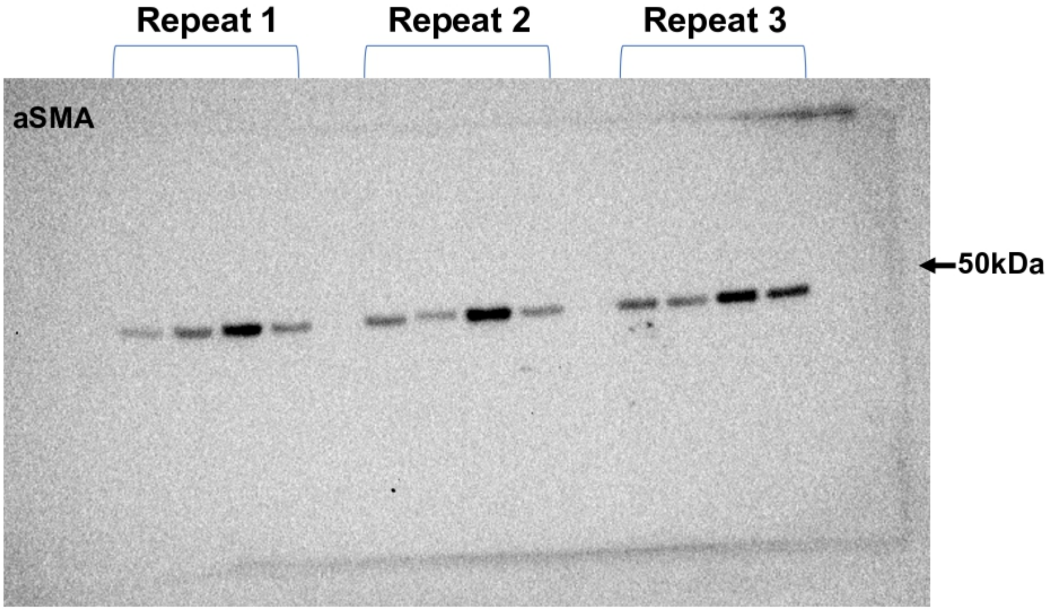

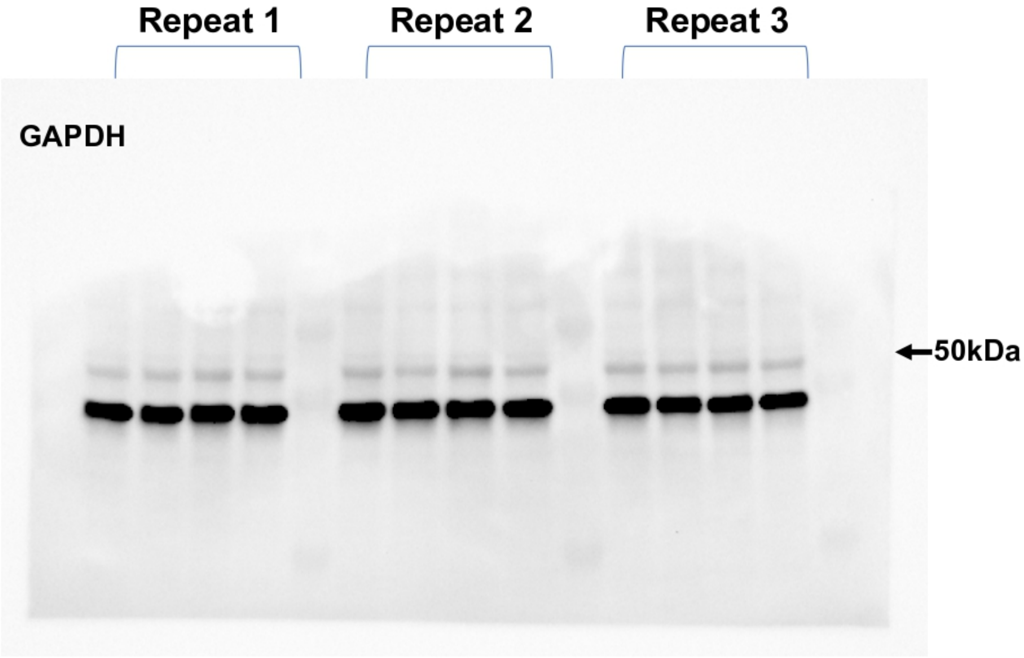

Supplementary Figure S9. Replicates of Western blot images from Figure 2A.

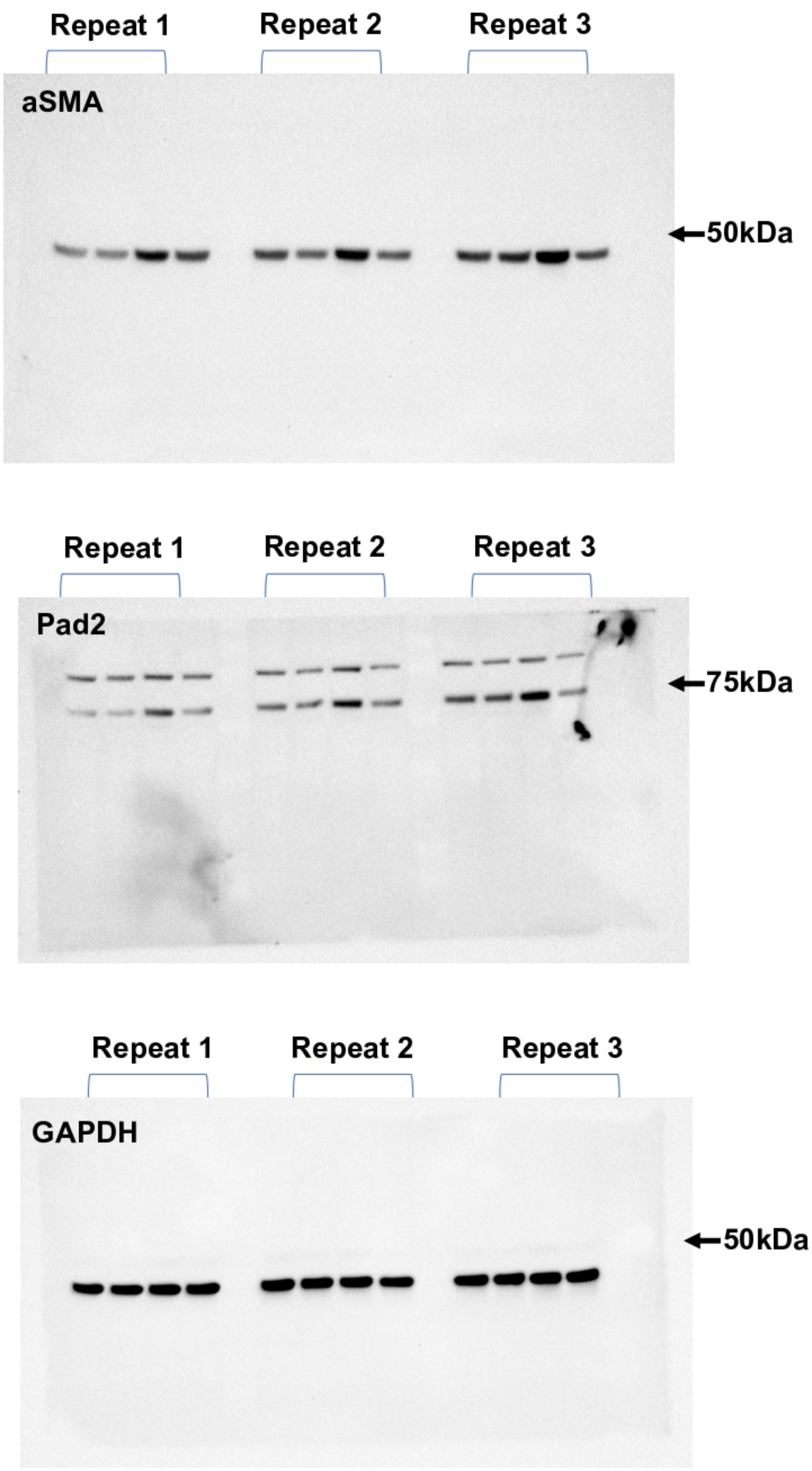

Supplementary Figure S9. Replicates of Western blot images from Figure 2B.

a)

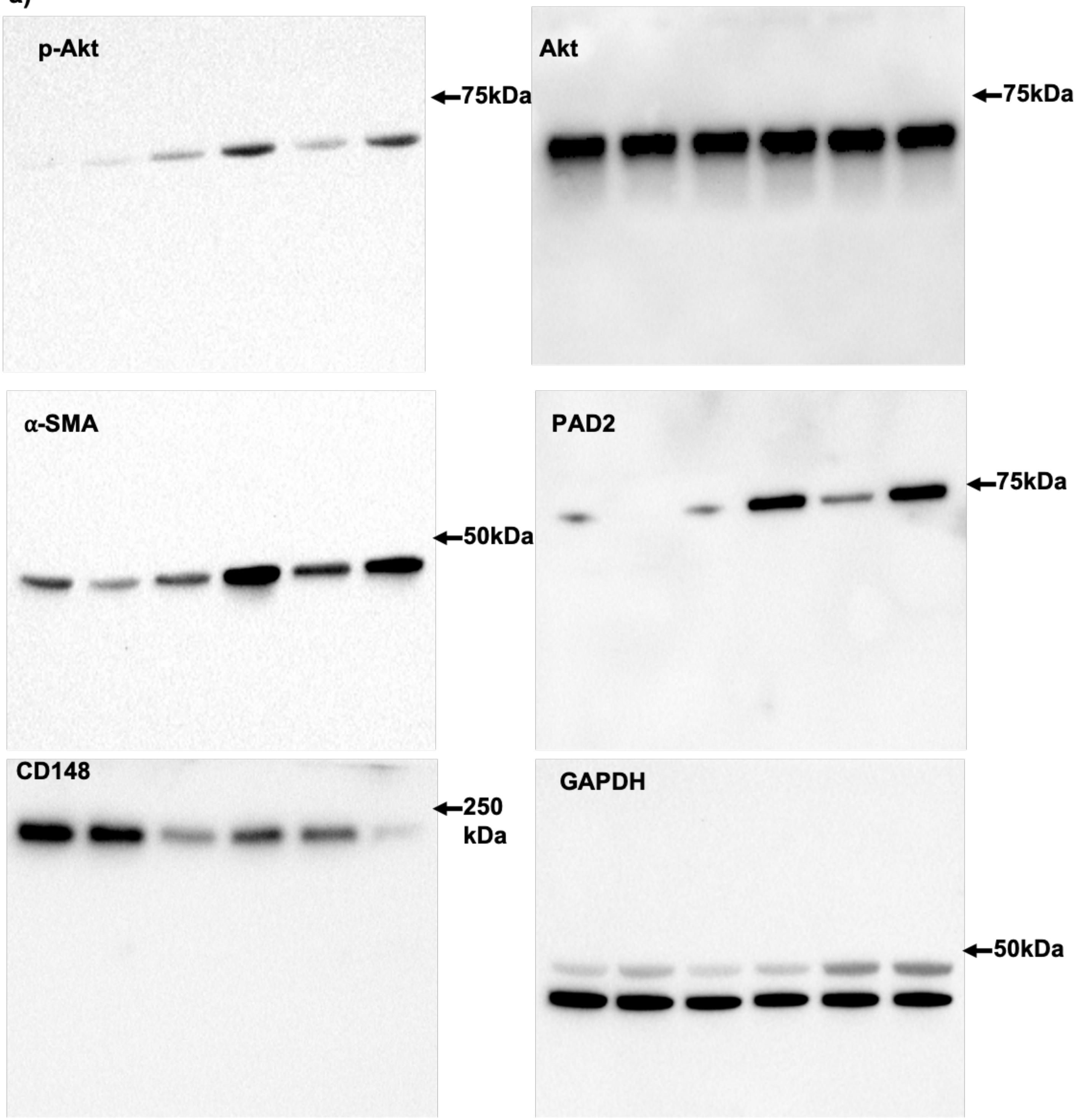

Supplementary Figure S10. Original Western blot images from Figure 3.

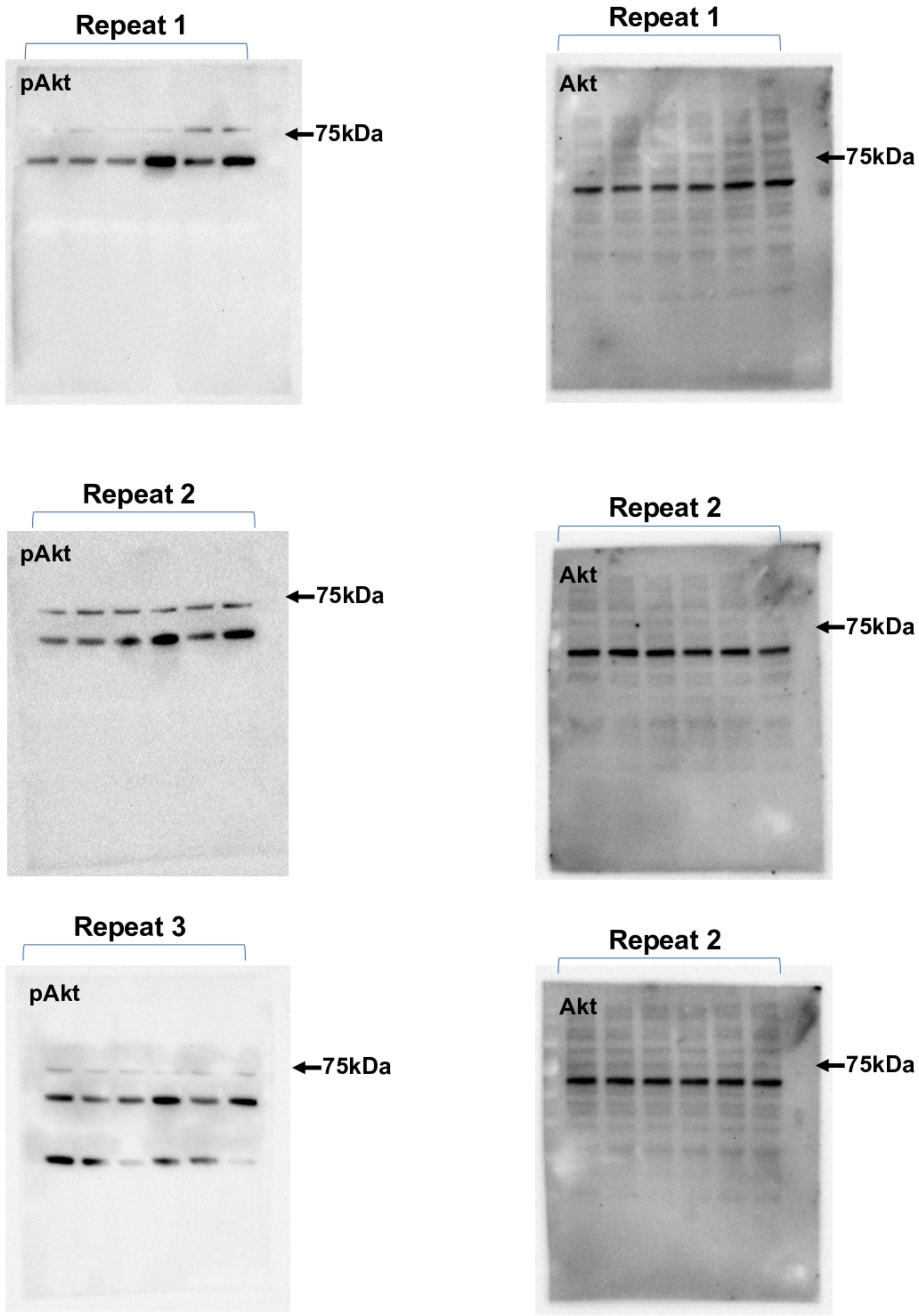

Supplementary Figure S10. Replicates of Western blot images from Figure 3A.

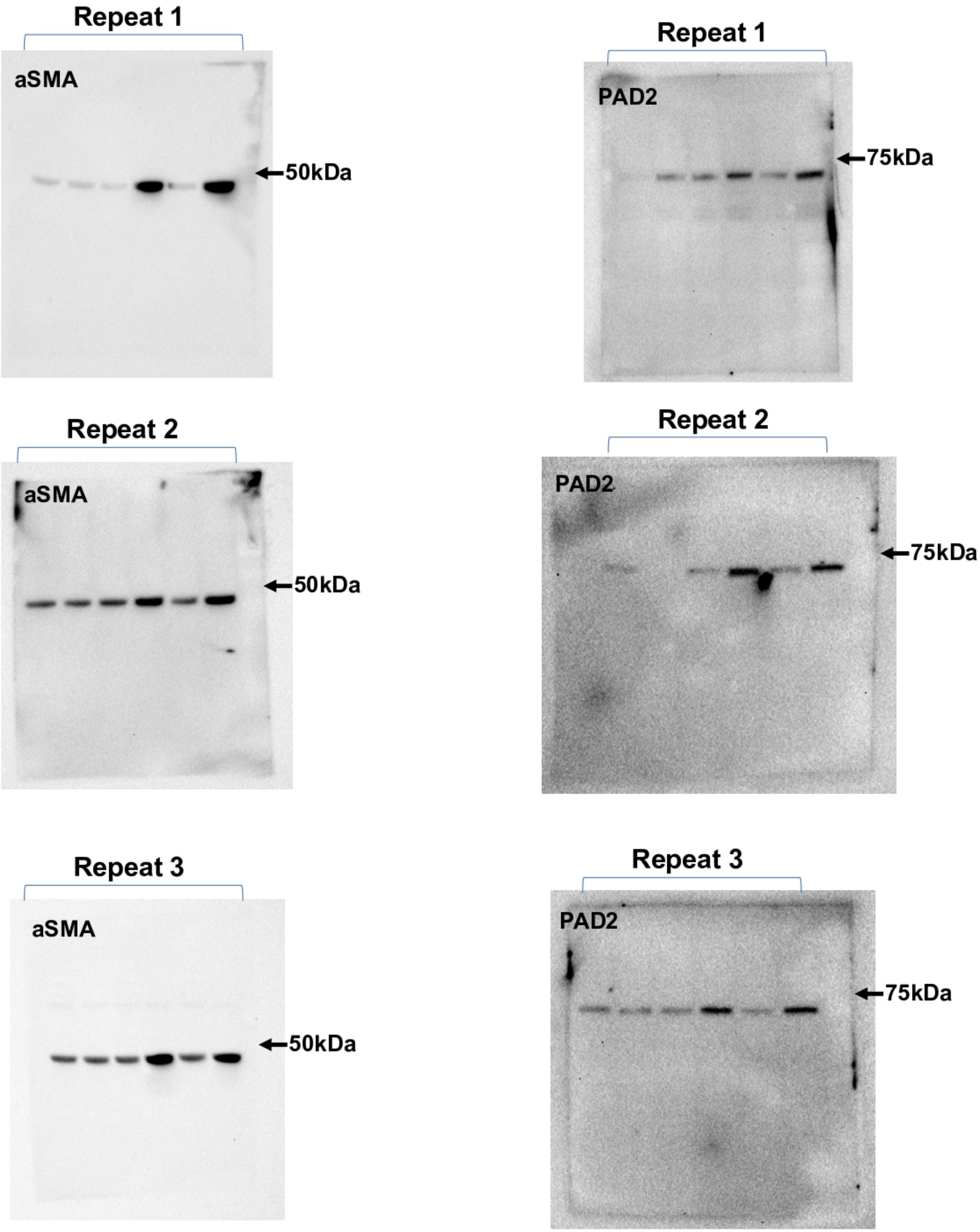

Supplementary Figure S10. Replicates of Western blot images from Figure 3A.

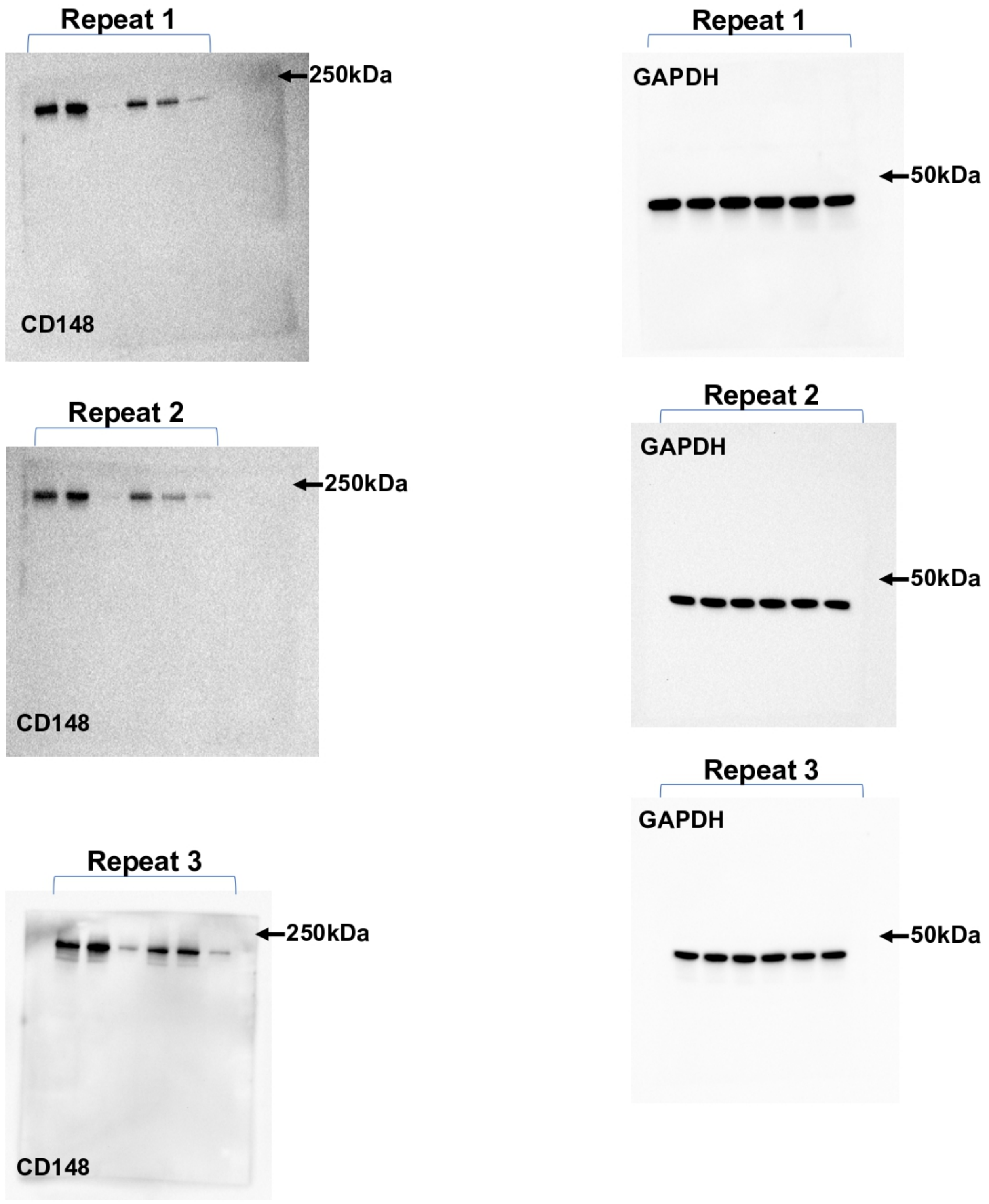

Supplementary Figure S10. Replicates of Western blot images from Figure 3A.

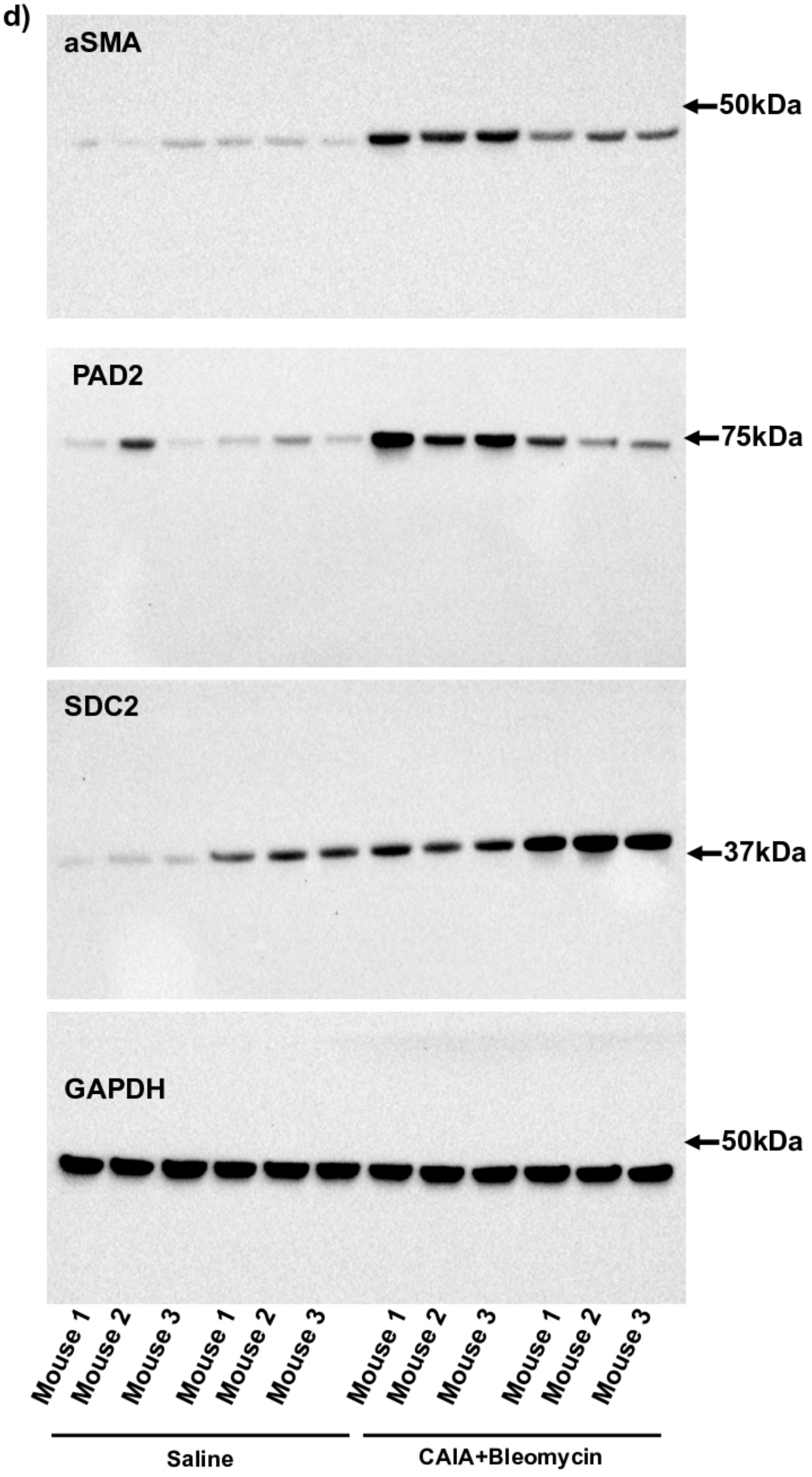

Supplementary Figure S11. Original Western blot images from Figure 4.

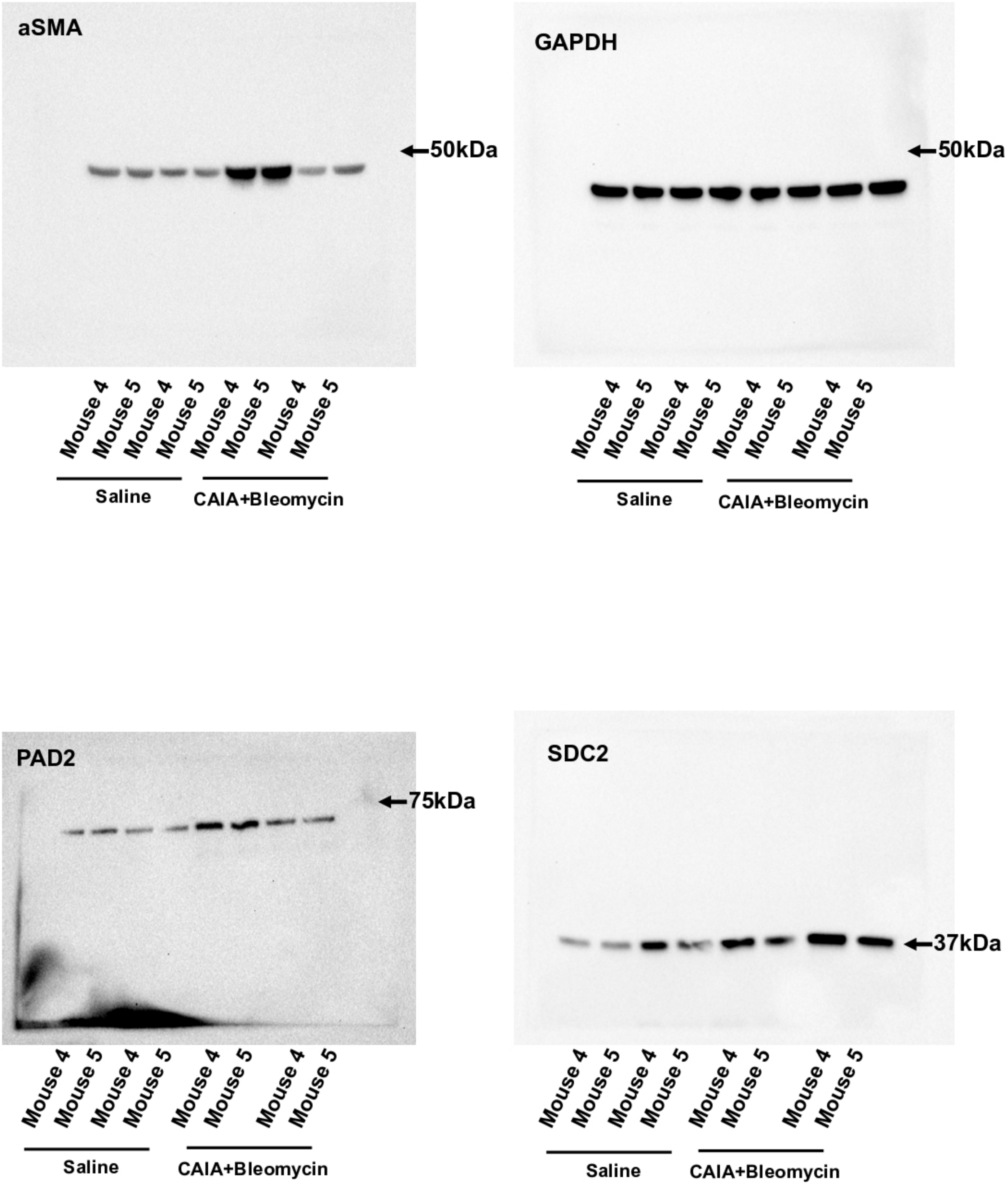

Supplementary Figure S11. Replicates of Western blot images from Figure 4.

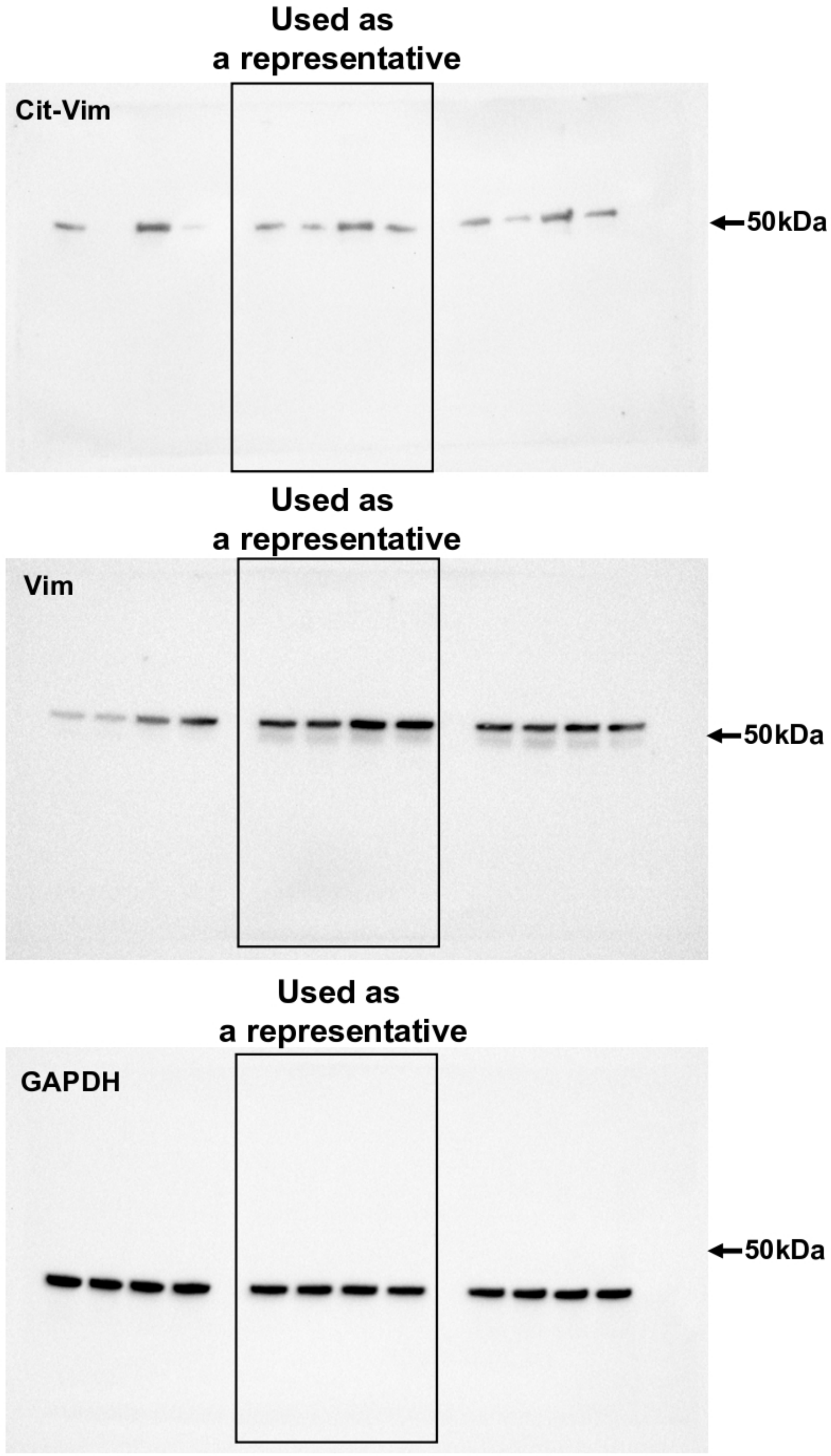

Supplementary Figure S12. Original Western blot images from Supplementary Figure S2.

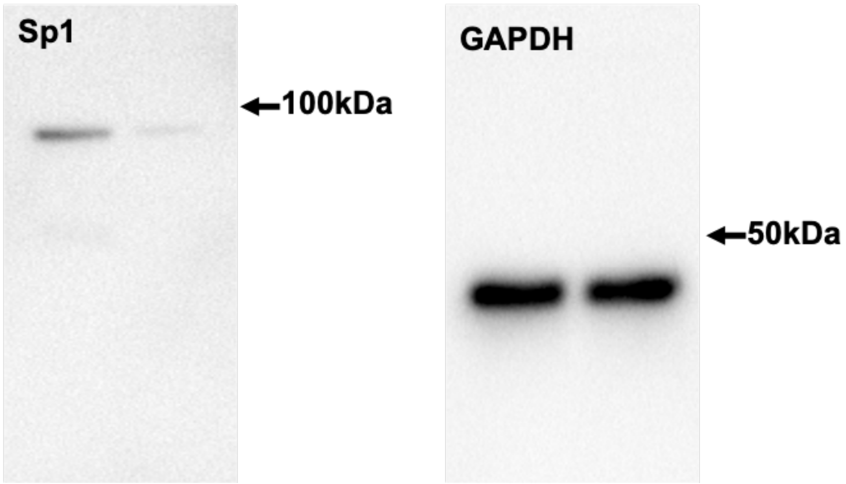

Supplementary Figure S13. Original Western blot images from from Supplementary Figure S5.

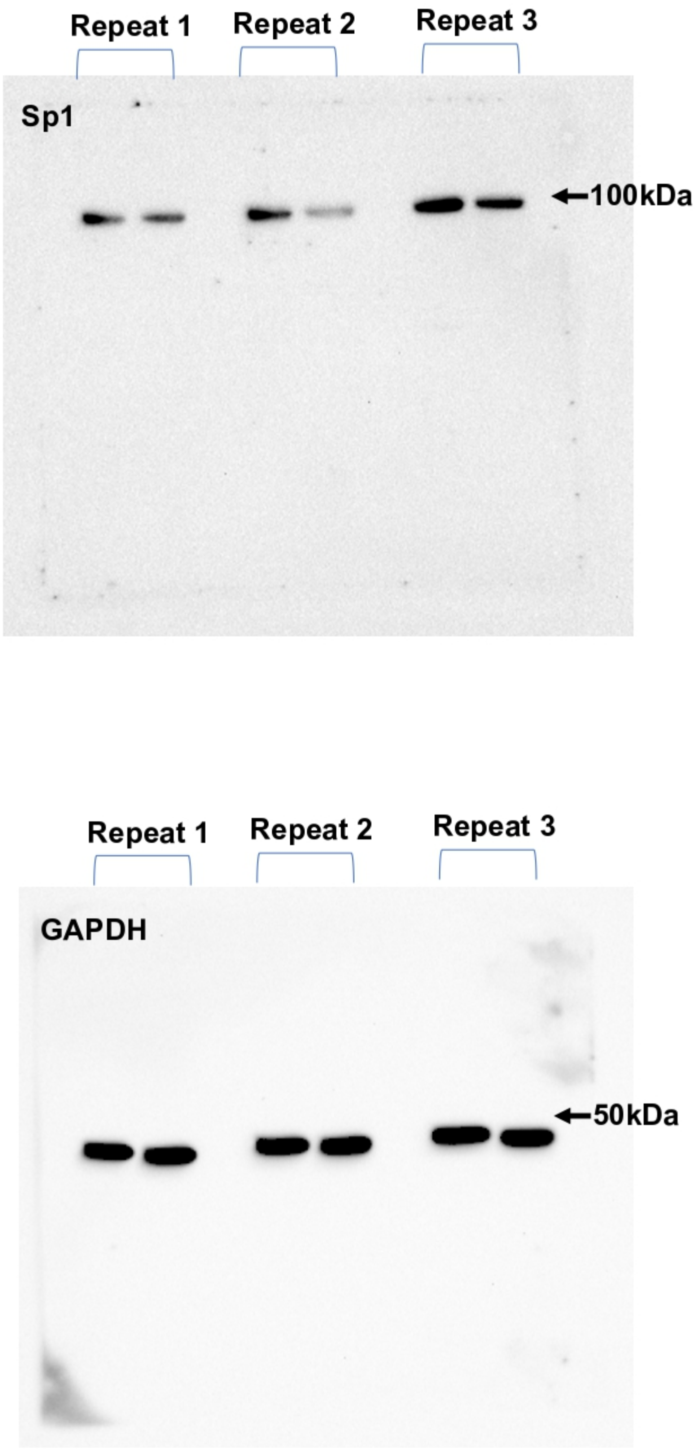

Supplementary Figure S13. Replicates of Western blot images from Supplemental Figure S5.

SUPPLEMENTARY TABLES

Supplementary Table S1. Primer sequences.

Gene	Forward (5′-3′)	Reverse (5′-3′)
Human GAPDH	GGATTTGGTCGTATTGGG	GGAAGATGGTGATGGGATT
Human PADI1	GAGTGATGGACACTCATGGC	CAGATGGTCAGCTTGCAGTT
Human PADI2	TCTCAGGCCTGGTCTCCAT	AAGATGGGAGTCAGGGGAAT
Human PADI3	AGCAATGACCTCAACGACAG	TGAGGTAGAGCACCGCATAG
Human PADI4	TCACCTACCACATCAGGCAT	CATGTTCCACCACTTGAAGG
Human PADI6	CAGGACACTGAGGACCATAAAG	GTACTCTTTCCCTTGGACCTTG
Human COL1A1	AAGGGACACAGAGGTTTCAGTGG	CAGCACCAGTAGCACCATCATTTC
Human FN	GCCAGATGTGAGCTGCAC	GAGCAAATGGCACCGAGATA
Mouse Gapdh	GTTGTCTCCTGCGACTTCA	GGTGGTCCAGGGTTTCTTA
Mouse Col1a1	ACCTCAGGGTATTGCTGGAC	CACCACTTGATCCAGAAGGA
Mouse Fn	ATGCAACGATCAGGACACAA	TGTGCCTCTCACACTTCCAC
Mouse Acta2	ATCACCAACTGGGACGACAT	CATACATGGCTGGGACATTG
Mouse Padi2	GAGGAGCGTTCTTCACTTTCT	GGCAGGAGCATCTTCATATACC

Supplementary Table S2. Abbreviations utilized in the text.

Abbreviation	Phrase Abbreviated
α-SMA	Alpha-smooth muscle actin
ACPA	Anti-citrullinated protein antibodies
Bleo	Bleomycin
CAIA	Collagen antibody-induced arthritis
Cit-vimentin	Citrullinated vimentin
COL1A1	Collagen 1a1 (gene symbol)
FN	Fibronectin (gene symbol)
IL-6	Interleukin-6
ILD	Interstitial lung disease
IPF	Idiopathic pulmonary fibrosis
LPS	Lipopolysaccharide
mTOR	Mammalian target of rapamycin
NF-κB	Nuclear factor kappa-light-chain enhancer of activated B cells
PAD2	Peptidylarginine deiminase 2
PI3K	Phosphoinositide 3-kinase
RA	Rheumatoid arthritis
RT-PCR	Real-time polymerase chain reaction
Scr	Scrambled
SDC2	Syndecan-2
shRNA	Small hairpin RNA
TGF-β1	Transforming growth factor beta-1
UIP	Usual interstitial pneumonia

SUPPLEMENTARY REFERENCES

1 Dimitrova, P. *et al.* Abrogated RANKL expression in properdin-deficient mice is associated with better outcome from collagen-antibody-induced arthritis. *Arthritis Res Ther*. **14**, R173 (2012).

2 Khachigian, L. M. Collagen antibody-induced arthritis. *Nat Protoc*. **1**, 2512-2516 (2006).

3 Tsoyi, K. *et al.* CD148 Deficiency in Fibroblasts Promotes the Development of Pulmonary Fibrosis. *Am J Respir Crit Care Med*. **204**, 312-325 (2021).
